# Prevalence of cryptosporidiosis and other enteric pathogens in calves in France: effects of rotavirus, coronavirus and *E. coli* vaccination and transmission routes

**DOI:** 10.1186/s13567-025-01609-6

**Published:** 2026-06-09

**Authors:** Damien Costa, Romy Razakandrainibe, Pierre Emilien Dorgebray, Venceslas Villier, Romain Coppée, Rachel Lefebvre, Sarah Barbey, Virginie Denizot, Pauline Martin, Tiffany Pezier, Sonia Lacroix-Lamande, Loïc Favennec, Fabrice Laurent

**Affiliations:** 1 Laboratory of Parasitology-Mycology, UR7510 ESCAPE, Univ Rouen Normandie, University Hospital of Rouen, 76000 Rouen, Normandie France; 2 INRAE, AgroParisTech, GABI, Université Paris‐Saclay, 78350 Jouy‐en‐Josas, France; 3 INRAE, UE326 Domaine Expérimental du Pin, 61310 Gouffern en Auge, France; 4 GDS Bretagne, Innoval, 35538 Noyal-sur-Vilaine, France; 5 INRAE, UMR1282 Infectiologie Et Santé Publique, 37380 Nouzilly, France; 6 National Reference Center for Cryptosporidiosis, Microsporidia and other digestive Protozoa, University hospital of Rouen, 76000 Rouen, Normandie France

**Keywords:** Cattle, pathogens, vaccination, *Cryptosporidium*, transmission

## Abstract

Newborn calf diarrhea is one of the most important diseases in bovine rearing, with various infectious origins. The aim of the study was to evaluate distribution of enteropathogens in calves, effect of vaccination and document transmission routes of cryptosporidiosis. In this study, 1159 stool samples were collected from calves and dams in France in: i) an experimental unit benefiting from high hygienic conditions and ii) farms selected on historical context of recurrent neonatal calf diarrhea. Stool samples were screened for enteric pathogens using the Speed-V-Diar-5^©^ antigen test (Virbac Diagnostics) and *Cryptosporidium* spp. were additionally identified by PCR, genotyped and subjected to multilocus variant analysis (MLVA). The effect of prepartum vaccination against bovine coronavirus, rotavirus and *E. coli* was evaluated. Results showed that *Cryptosporidium* was the most frequent enteropathogen, followed by bovine rotavirus. Only 16% of calves were symptomatic when systematic vaccination of dams was performed. The Speed-V-Diar-5^©^ antigen test (Virbac Diagnostics) achieved sensitivities for *Cryptosporidium* detection ranging from 67 to 70% in symptomatic calves depending of sites. For the first time, *C. mortiferum* and *C. meleagridis* were reported in symptomatic calves. A large diversity of *C. parvum* genotypes was identified in calves, only from the zoonotic families IIa and IId. The IIaA15G2R1 genotype represented the large majority of identified genotypes. The comparison of genotypes between calves and dams suggest that vertical transmission was not the primary route of transmission. Calf-to-calf transmission and environmental contamination probably play important roles in the spread of *Cryptosporidium* among calves.

**Supplementary Information:**

The online version contains supplementary material available at 10.1186/s13567-025-01609-6.

## Introduction

Diarrhea commonly affects newborn calves and is a major cause of productivity and economic loss for cattle producers worldwide. Economic losses are multifactorial and include costs of treatment, management of enteritis, reduced feed conversion, reduced production efficiency and losses due to animal mortality [1]. Neonatal diarrhea accounts for over 25% of farm visits and is the leading cause of mortality and stunted growth in calves under one month of age. Fatal cases are due to dehydration and acidosis resulting in anorexia and ataxia in calves [2, 3]. The causes of diarrhea can be either infectious or non-infectious. Non-infectious causes include vitamin deficiencies, dietary changes, or errors in milk replacer feeding [4]. Several enteric pathogens have been implicated, including bacteria, viruses, and parasites. *Cryptosporidium* sp., bovine coronavirus*,* bovine rotavirus, *E. coli* and *Salmonella* spp. are commonly detected in calves [5]. Vaccination of pregnant dams may be recommended to limit infections in calves. Ideally, calves should receive colostrum from their dams to provide passive immunization [6]. Only one vaccine currently exists to prevent cryptosporidiosis based on maternal immunity and was recently announced: the “Bovilis Cryptium” (MSD^©^).

*Cryptosporidium* is an intracellular protozoan parasite responsible for cryptosporidiosis in a variety of vertebrate hosts, including humans [7–9]. *Cryptosporidium* oocysts are shed in the feces of infected hosts. Infection occurs by ingestion of oocysts via the fecal–oral route, either directly – through contact with contaminated feces – or indirectly – through environmental contamination. The first case of calf infection was published in 1971 in an 8-month-old female who was anorectic, weak, and ataxic [1, 10]. Studies have shown that young calves infected with *Cryptosporidium parvum* experience profuse watery diarrhea, inappetence, lethargy, dehydration, and in severe cases, death [9, 11, 12]. Depending on the infectious dose, symptoms usually appear 3–4 days after oocyst ingestion and persist for one to two weeks [1]. Several detection methods exist (microscopy, enzyme-linked immunosorbent assay, molecular methods…) with different reported performances [13]. Cryptosporidiosis is now recognized as one of the most common diarrheal diseases in calves. Depending on the case, oocyst shedding can continue for 4 to 12 days post-infection [1, 14]. An infected calf can shed more than 1 × 10^10^ oocysts per day [15]. In newborn calves, ingestion of 17 oocysts has been shown to be sufficient to cause diarrhea [14]. More than 44 *Cryptosporidium* species have been described, infecting a wide variety of hosts. Currently, 21 species have been reported in humans but *C. parvum* and *C. hominis* are responsible of 95% of human infections [16]. Zoonotic features of *C. parvum* result in numerous cases of human infections [17–20]. The distribution of *Cryptosporidium* species in cattle is thought to be age-dependent [1, 21, 22]. Newborn calves are frequently infected with *Cryptosporidium* spp., but the source of contamination remains undefined. Reducing *Cryptosporidium* environmental contamination is challenging due to the extensive shedding of oocysts and their high resistance to chlorine. Therefore, improved knowledge of the distribution of *Cryptosporidium* species and potential transmission routes are essential to manage the associated infectious risk.

This study aims to address significant gaps in our understanding of calf diarrhea with a focus on diagnostic and preventive measures. Our specific objectives are: (i) to determine the distribution of enteropathogens (bovine rotavirus, bovine coronavirus, *E. coli*, *Cryptosporidium*) in studied calves; (ii) to evaluate the effect of vaccination on symptomatology of calves and on the distribution of enteropathogens; (iii) to evaluate the performance of an antigen detection method for cryptosporidiosis in symptomatic and asymptomatic calves; (iv) to analyze the distribution of *Cryptosporidium* species in cattle.

We found that *Cryptosporidium* was predominant among the studied enteropathogens. Systematic vaccination seems to have a beneficial effect on symptom expression. Antigenic tests are relevant for *C. parvum* detection when animals are symptomatic. A large diversity of *Cryptosporidium* species and genotypes was observed: the IIaA15G2R1 hypertransmissible ones being the most represented. Some data on transmission of *Cryptosporidium* subtypes were described.

## Materials and methods

### Sampling sites and stool collection

Sampling of animals took place at an experimental unit of the National Institute for Agricultural Research and at farms (*N* = 33) affiliated to the Brittany Health Defense Group (GDS). Sampling was conducted annually from November to January from 2018 to 2021. The INRAE experimental unit has a large herd of about 260 calves per year, of which 150 are Holstein cows. Calving took place throughout the year, with an increased calving season in February and March. The INRAE site should be considered as a highly supervised unit without important microbial pressure. For the GDS sampling sites, a total of 33 farms were sampled for the duration of the study and were initially selected based on the recurrence of neonatal pathology (both mortality and morbidity). A total of 593 stool samples were collected from calves (241 from “INRAE” and 352 from “GDS”) and 566 from cows (194 from “INRAE” and 372 from “GDS”). Additionally, data were collected on farm origin, filiation, vaccination status and symptomatology. Data not fully completed and data reported as doubtful (antigen test results) were excluded. Symptomatology was defined as sudden onset of diarrhea, dehydration or weakness and was initially identified by farm workers. Calves were examined daily by farm workers from 0 to 14 days of age. Each week, veterinarians systematically sampled stools from newborn calves (since the previous week) and additionally record clinical status of calves (i.e. good condition or diarrhoea, dehydration, weakness) and confront it with symptomatology reported by farm workers. The following week, symptomatology of calves were also recorded by veterinarians. Considering both farm workers and veterinarians investigations, calves that have not suffered from diarrhoea, dehydration or weakness during this 2 weeks period were considered asymptomatic. If calves were symptomatic during any of these 2 weeks, they were considered symptomatic. Calves were separated from their dams at most 24 h after birth and housed: (i) in individual pens for at least 14 days at “INRAE” site; (ii) in individual pens or small groups (≤ 5 calves) at GDS sites. Stool samples were collected on calves from one to 14 days of age (93% between 6 to 11 days of age) during calf handling and tested for enteropathogens (bovine rotavirus, bovine coronavirus, *E. coli* F5, *E. coli* CS31A, and *Cryptosporidium parvum*) using the Speed-V-Diar 5^©^ (Virbac Diagnostics) antigen test according to the manufacturer’s recommendations. Since sampling was systematically performed on each newborn calf before 14 days of age, farms were visited many times during the period of the study. Each calf was sampled only one times. Stool samples were then sent (+ 4 °C) to the National Reference Center for cryptosporidiosis for *Cryptosporidium* DNA testing, genotyping and multilocus variable number tandem repeat investigations.

### DNA extraction

Total DNA was extracted using the QIAamp PowerFecal DNA Kit (Qiagen, courtaboeuf, Hilden, France) following the manufacturer’s instructions as previously published [23]. Briefly, 100 µL of total DNA was obtained after mechanical pretreatment (bead beating) followed by sequential purification steps including column purification.

### Screening for enteric pathogens

DNA samples were analyzed by molecular methods for species identification and subtyping. Species identification was performed by sequencing the *SSU* rRNA gene as described by Koehler et al. [24]. Briefly, primary PCR was performed using the following primers: XF2 5′-GGAAGGGTTGTATTTATTAGATAAAG-3’ and XR2 5′-AAGGAGTAAGGAACAACCTCCA-3′ followed by a second PCR using pSSUf 5′-AAAGCTCGTAGTTGGATTTCTGTT-3’ and pSSUr 5′-ACCTCTGACTGTTAAATACRAATGC-3’. For the first PCR, the cycling protocol started with an initial denaturation at 94 °C for 5 min. This was followed by 30 cycles consisting of denaturation at 94 °C for 45 s, annealing at 45 °C for 2 min, and elongation at 72 °C for 1.5 min. A final extension step was performed at 72 °C for 10 min. For the second PCR, the cycling protocol started with an initial denaturation at 94 °C for 5 min. This was followed by 35 cycles of denaturation at 94 °C for 30 s, annealing at 55 °C for 30 s, and elongation at 72 °C for 30 s. A final extension was performed at 72 °C for 10 min. Amplicons were purified using Exosap-IT (Thermo Fisher Scientific, France) and sequenced unilaterally (BigDye^®^ Terminator v.3.1 chemistry, Applied Biosystems, Illkirch, France) using the reverse primers used in the secondary (nested) PCR. Each sequence obtained was compared with the non-redundant database of NCBI using BLASTn searches (default parameters).

*gp60* subtypes were identified according to the protocol described by Sulaiman et al. [25]. A nested PCR was performed with the following primers: AL3531 F (5’ -ATAGTCTCCGCTGTATTC-30) / AL3533 R (5’ -GAGATATATCTTGGTGCG-3’) and secondly AL3532 F (5’ -TCCGCTGTATTCTCAGCC-3’) / LX0029 R (5’ -CGAACCACATTACAAATGAAGT-3’). The cycling procedure started with an initial denaturation at 94 °C for 3 min, followed by 40 cycles consisting of denaturation at 94 °C for 45 s, annealing at 54 °C for 45 s, and elongation at 72 °C for 60 s. A final extension step was performed at 72 °C for 7 min. Sequencing was performed on an AB3500 automated sequencer (Applied Biosystems, Illkirch, France).

Multilocus variable number tandem repeat analysis (MLVA) was performed on *gp60* identical *C. parvum* subtypes according to Risby et al. [26]. The following loci were analyzed: *cgd1; cgd4*; *MSF*; *cgd5*; *cgd6*, *cgd8* and *MM19*. A MLVA profile was created by merging the calculated number of repeats of each locus.

### Statistical analyses

Statistical analyses were performed using chi-squared or Student’s *t*-tests depending on the conditions studied. Details are presented in Figures. Results with a *p*-value < 0.05 were considered as significant.

## Results

### Pathogen prevalence and symptomatology

**Figure 1:**
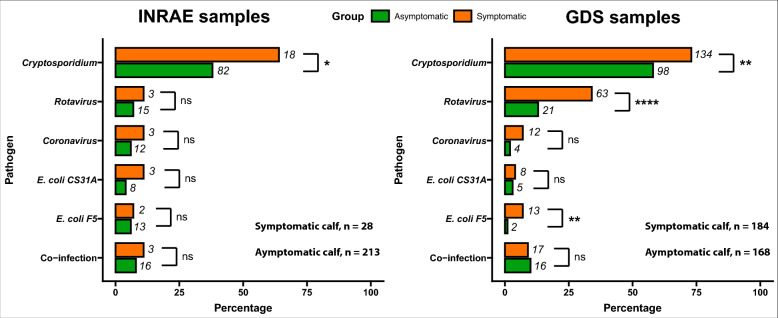
**Distribution of enteropathogens according to symptomatology of calves and sampling sites.** Results were obtained using the Speed-V-Diar 5© (Virbac Diagnostics) antigen test except for *Cryptosporidium spp.* where PCR were used to detect the parasite. Student *t* test was performed to compare prevalence of pathogens accordingly to symptomatology. *p-value ≤ 0.05, **p value ≤ 0.01, ***p-value ≤ 0.001 and ****p-value ≤ 0.0001.

Regarding occurrence of each digestive pathogen studied, *Cryptosporidium* was the most frequent in both INRAE (64%; 18/28 symptomatic calves) and GDS sites (73%; 134/184 symptomatic calves); followed by rotavirus (11%; 3/28 symptomatic calves) in INRAE and 34% (63/184 symptomatic calves) in GDS sites. Asymptomatic carriage was also observed in some cases (38% for *Cryptosporidium*; 82/213 asymptomatic calves in INRAE; 58% for *Cryptosporidium*; 98/168 asymptomatic calves for GDS) (Figure 1 and Additional file 1). Mixed infections were observed in 11% in symptomatic calves in INRAE and 9% in GDS sites (Additional file 1). No correlation (chi-squared test) between pathogens was observed, indicating that the presence of one pathogen does not significantly influence infection with another opportunistic pathogen.

### Effects of vaccination

**Figure 2:**
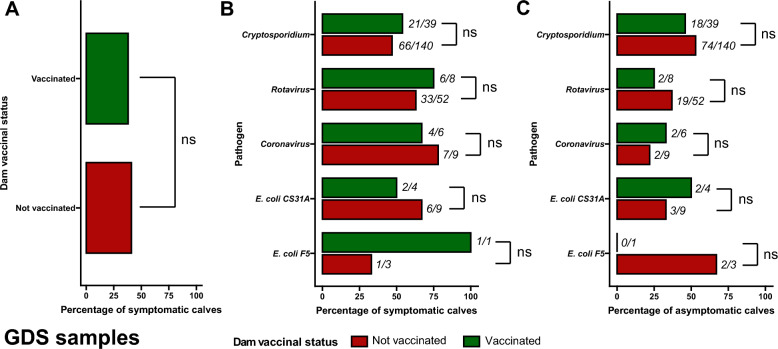
**Effect of vaccination on symptomatology in calves according to detected enteropathogens.** **A**: Proportion of symptomatic calves according to dams vaccination. **B**: Distribution of enteropathogens in symptomatic calves according to dams vaccination. **C**: Distribution of enteropathogens in asymptomatic calves according to dams vaccination.

A total of three hundred and thirty-seven calves were born to cows vaccinated with Trivacton^©^ (Boehringer Ingelheim Animal Health), Rotavec^©^ (MSD) or Bovigen^©^ (Virbac), all of which target bovine coronavirus, rotavirus and strains of *E. coli.* For INRAE, all dams were vaccinated whereas for GDS, only 25% (76/300) of calves born from vaccinated dams. Regarding GDS, the proportion of symptomatic calves born from vaccinated dams was of 38% (29/76) versus 41% (92/224) when dams were not vaccinated (Figure 2A). No significant difference in prevalence of enteropathogens was observed according to dam vaccinal status, (Figure 2B and C). Regarding INRAE (with 100% of dams vaccinated), the proportion of symptomatic calves was of 16% (38/205). Proportion of enteropathogens according to symptomatology in shown in Figure 1.

### Sensitivity, specificity, positive and negative predictive values evaluation for ***Cryptosporidium*** detection using the Speed-V-Diar 5^©^ antigen test

Parameters of the Speed-V-Diar 5^©^ antigen test (Virbac Diagnostics) (using PCR as reference method) for the detection of *Cryptosporidium* are presented in Table 1. Interestingly, the Speed-V-Diar 5^©^ antigen test (Virbac Diagnostics) was always negative for *C. bovis* (*N* = 11 for both symptomatic and asymptomatic calves) from the INRAE site but was positive for 2 *C. bovis* in symptomatic calves from GDS sites and negative for 2 *C. bovis* in asymptomatic calves from GDS sites (Additional file 1). The antigen test was negative for: *C. ryanae* (*N* = 1 in an asymptomatic calf from INRAE site), for *C. mortiferum* (*N* = 1 in a symptomatic calf from GDS sites) and for *C. andersoni* (*N* = 2 in asymptomatic calves from GDS sites). The antigen test was positive for *C. meleagridis* (*N* = 1 in a symptomatic calf from GDS sites. (Additional file 1).

**Table 1:** **Performances in terms of sensitivity, specificity, positive and negative predictive values of the Speed-V-Diar 5**^©^ **(Virbac Diagnostics) antigen detection method for** ***Cryptosporidium***

	Symptomatic calves		Asymptomatic calves	
	INRAE	GDS	INRAE	GDS
True Positive	18	94	13	20
False Positive	2	19	12	8
True Negative	9	30	114	63
False Negative	9	40	69	78
Sensitivity (%)	67 (18/27)	70 (94/134)	16 (13/82)	20 (20/98)
Specificity (%)	82 (9/11)	61 (30/49)	90 (114/126)	89 (63/71)
Positive predictive value (%)	90 (18/20)	83 (94/113)	52 (13/25)	71 (20/28)
Negative predictive value (%)	50 (9/18)	43 (30/70)	62 (114/183)	45 (63/141)

PCR was used as the reference method for calculation. True positive are defined as both positive antigenic test and PCR. True negative are defined as both negative antigenic test and PCR. False positive are defined as positive antigenic test but negative PCR. False negative are defined as negative antigenic test but positive PCR

### Distribution of *Cryptosporidium* species

*C. parvum* was the predominant *Cryptosporidium* species under the studied conditions. For the “INRAE” sampling site, 101 *Cryptosporidium* isolates from calf samples were successfully identified (all positive isolates were successfully identified): 86% (87/101) of *C. parvum*, 11% (11/101) of *C. bovis*, 2% (2/101) of *C. hominis* and 1% (1/101) of *C. ryanae*; and 66 *Cryptosporidium* isolates from cow samples were successfully identified: 83% (55/66) of *C. parvum*, 12% (8/66) of *C. bovis*, 1% (1/66) of *C. hominis, 1*% (1/66) of *C. andersoni*, and 1% (1/66) of *C. ryanae.* 31 *Cryptosporidium* positive isolates from cows could not be identified due to low parasite concentration (Additional file 3).

**Figure 3:**
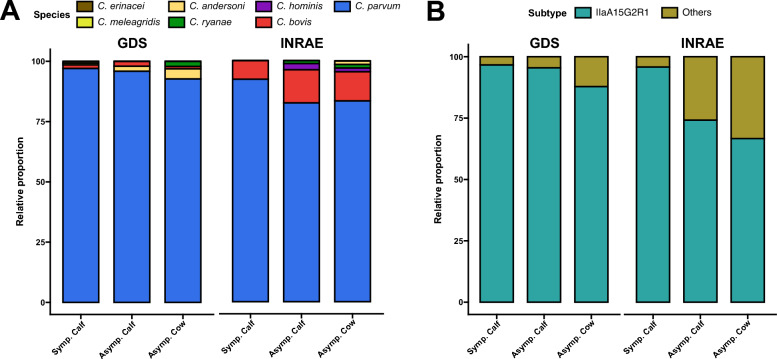
**Distribution of** ***Cryptosporidium*** **species and gp60 genotypes accordingly to sampling sites, symptomatology and animals**. Symp.: symptomatic; Asymp.: asymptomatic.

For the “GDS” sampling site, 232 *Cryptosporidium* isolates from calf samples were successfully identified (all positive isolates were identified): 97% (224/232) of *C. parvum*, 2% (4/232) of *C. bovis*, 1% (2/232) of *C. andersoni*, < 1% (1/232) of *C. mortiferum* and < 1% (1/232) of *C. meleagridis*; and 96 *Cryptosporidium* isolates from cow samples were successfully identified: 93% (89/96) of *C. parvum*, 4% (4/96) of *C. andersoni*, 2% (2/96) of *C. ryanae* and 1% (1/96) of *C. bovis*. 32 *Cryptosporidium* positive isolates from cows could not be identified due to low parasite concentration (Additional file 3). The IIaA15G2R1 genotype was fully predominant for both INRAE and GDS (Figure 3 and Additional files 1, 2 and 3)*.*

### Distribution of *Cryptosporidium* spp. in calves and dams

**Figure 4:**
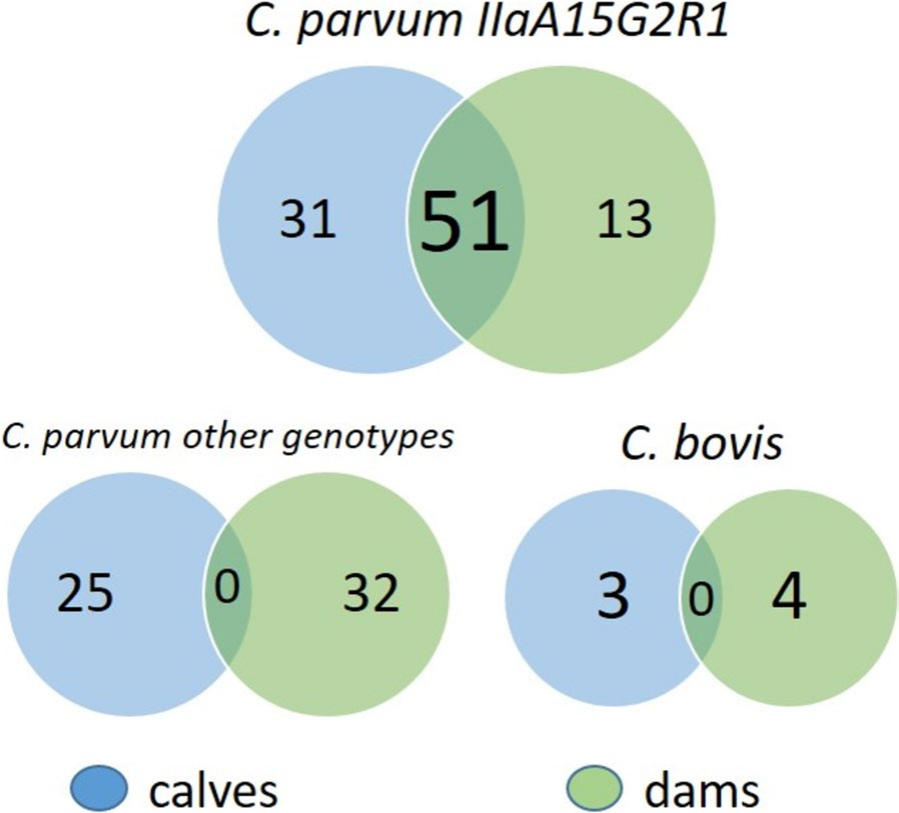
**Venn diagram showing the distribution of** ***Cryptosporidium spp.*** **between calves and their dams**.

For INRAE, 43 *Cryptosporidium* spp. genotypes were identified in calves whereas no *Cryptosporidium* spp. DNA was detected from corresponding dams. For GDS, 148 *Cryptosporidium* spp. genotypes were identified in calves whereas no *Cryptosporidium* spp. DNA was detected from corresponding dams. For INRAE, 23 different *Cryptosporidium* species or *gp60* genotypes were identified and 13 for GDS. A total of 95 genotypes could be compare (fully identified) between calves and corresponding dams (37 for INRAE and 58 for GDS): except for the IIaA15G2R1 genotype, no match was observed between genotypes observed in calves and dams (Figure 4 and Additional file 3). Due to high diversity in IIaA15G2R1 strains, additional analyses on several loci (in addition to *gp60*) were required to confirm or infirm matches between calves and dams. The MLVA profiles of the dams were either incomplete or could not be determined due to low DNA concentration. When available, the incomplete MLVA profiles of the dams (4–12-X-X-X-32/29–15) showed deviation from those of their calves (4–12-5–8-27–32-16).

**Figure 5:**
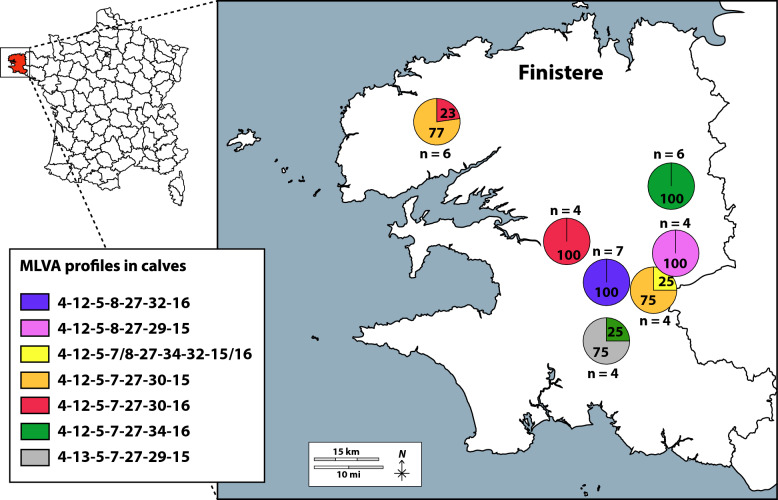
**Distribution of** ***C. parvum*** **IIaA15G2R1 MLVA profiles on 7 farms in Finistère, France**. Pie charts represent the proportions of each MLVA profile within each farm.

To evaluate distribution of *C. parvum* IIaA15G2R1 strains in the environment, the distribution of *C. parvum* IIaA15G2R1 MLVA profiles according to farms localization is shown in Figure 5. Only one or two different MLVA profiles were observed per farm and the profiles were closely related but generally farm specific suggesting clonal contamination per farm in the studied conditions and transmission to calves via the local environment.

## Discussion

### Pathogens prevalence

In accordance with the literature, *Cryptosporidium* was the most frequent pathogen in the studied population. Already published data showed a prevalence of cryptosporidiosis ranging from 17.9% to 88%, depending on the symptoms exhibited by the cattle and the farms studied [8, 27, 28]. In symptomatic calves, rotavirus was frequently reported as the second most common infection, with a prevalence ranging from 6.37% to 14.3%. Bovine coronavirus and *E. coli* infections were considered negligible [29–32]. Such variations in literature can be explained by methodology used (microscopy, antigen detection, PCR) and sampling. PCR method is the most sensitive but DNA could be detected several days after the end of infection. It has been reported that *Cryptosporidium* spp. is a risk factor for rotavirus, and vice versa [31, 32]. However, no significant co-infection was observed in our study.

### Effects of vaccination

Vaccinating very young animals may not be effective because their immune systems are not fully developed, or because maternal antibodies may interfere with the vaccine. Therefore, most studies have evaluated vaccination of pregnant cows on calf symptomatology. Variable efficacy depending on vaccine and pathogen has been reported [4, 33, 34]. Our study emphasized the effectiveness of using a multivalent vaccine to reduce symptoms in calves. Whatever the studied enteropathogen, no significant difference was observe in our study in observed symptomatology between calves born from vaccinated dams or not (GDS sites). A dedicated study should be done including more effective per studied enteropathogens to evaluate effect of vaccination. Overall proportion of symptomatic calves was significantly lower in INRAE (16% vs 40%, *p* < 0.0001) than GDS. Systematic vaccination seems to be interesting to reduce overall calves symptomatology. It should be confirmed using systematic vaccination on other farms, since the experimental unit feature of INRAE may bias this result. At the time of our study, no vaccine was commercially available for cryptosporidiosis. In April 2024, it was announced the commercialization of a cryptosporidiosis vaccine (Bovilis Cryptium® MSD). The vaccine targets the GP40 glycoprotein and must be administered to pregnant cows and passive immunity will be transferred via colostrum. Clinical impact should be evaluated in the future to complement our current observations.

### Performance of the studied antigen detection method

The symptomatology of the calves differed between the sampling sites. The sampling site “INRAE” is an experimental farm with important hygienic measures, which probably explains the observed low symptomatology of the calves. In contrast, the sampling site “GDS” consisted of farms in the Brittany region, selected because of the recurrence of neonatal digestive pathology in recent years. It was therefore important to analyze these two conditions independently. The manufacturer reported sensitivities of 93.5%, 94.3%, 97.2%, 93.5%, and 89% for rotavirus, coronavirus, *C. parvum*, *E. coli* F5, and *E. coli* CS31A, respectively. The observed *Cryptosporidium* sensitivities were lower than those reported by the manufacturer. However, species other than *C. parvum* were detected: *C. bovis* (not systematically), and *C. meleagridis*. However, for rare species, numbers were too small to draw solid conclusions. Under practical conditions, antigen tests provide good specificity for asymptomatic calves and acceptable sensitivity in symptomatic animals with the advantage of a rapid response time. However, PCR detection is more appropriate for epidemiologic studies.

### Distribution of *Cryptosporidium* species

It has been published that *C. parvum*, *C. andersoni*, *C. bovis* and *C. ryanae* are commonly detected in cattle worldwide: *C. parvum* clearly dominates (> 90%), especially in preweaned calves [21, 35, 36]. Contact with adult animals has been identified as a risk factor for *C. andersoni* and *C. bovis* infection in calves [35, 37]. *C. meleagridis* has been isolated primarily from birds and can infect humans [18, 38, 39]. The ability of *C. meleagridis* to infect calves has been demonstrated experimentally [40, 41], but *C. meleagridis* has rarely been reported in calves worldwide [38]. Infection of calves with *C. hominis* has been reported previously [7]. However, this is the first time that *C. mortiferum* has been detected from symptomatic calves. Interestingly, *C. mortiferum* and *C. meleagridis* were detected only in the sampling site “GDS”. *C. hominis* was detected only in the sampling site “INRAE”. Due to the characteristics of the experimental unit, animals from the “INRAE” sampling site were more exposed to humans than those from “GDS”, and conversely, animals from “GDS” sampling site were more exposed to wildlife than those from “INRAE”. Numbers were too small to affirm it but it could be linked to the observed rare species distribution.

### Evaluation of vertical transmission of *Cryptosporidium* spp.

Even if this study was not specifically designed to investigate vertical or horizontal transmission of contamination between calves and dams, some data were obtained to discuss it. Ideally, sampling of dams should be done the day of calving to investigate vertical transmission. In humans, *Cryptosporidium* DNA can still be detected between one to three months after contamination (experience of the French National Reference Center for Cryptosporidiosis). In this study, dams were sampled the same day of calves and sampling of calves was done in their first week of life. During a one week period, we can assume that *Cryptosporidium* DNA could be detected enabling investigation of vertical transmission.

Results shown a large diversity of *Cryptosporidium* genotypes in animals*.* Many calves were infected by *Cryptosporidium* spp. while dams not (in both INRAE and GDS)*. Cryptosporidium* species and genotypes distribution between calves and dams was different except for the most prevalent IIaA15G2R1 *gp60* genotype. Further analyses were necessary on supplemental loci using MLVA. Unfortunately, results are difficult to interpret due to low contamination of dams. For detection of low *Cryptosporidium* concentrations, the use of a specific method from cow samples should be used. It has been reported that performed acid flocculation + salt flotation (resulting in investigation of a larger starting sample ≈50 g) from cow samples before proceed to DNA extraction and amplification, strongly improve sensitivity of detection [42]. Using this method, Wells et al. have reported a systematic higher prevalence of *Cryptosporidium* in adult cows versus calves [43]. For studies focusing on vertical/horizontal route of transmission investigation, we recommend to take it into consideration. In absence of such specific pre-treatment on cows samples in our study, we probably underestimate frequency of *Cryptosporidium* in adult cows and limit genotypes comparison. The only partially interpretable MLVA profile appeared slightly different between dams and calves but effective were low. It seems more in favor of horizontal transmission in calves or eventually of host adaptation. MLVA profiles appeared mainly unique among calves from the same farm. MLVA profiles appeared to be close but almost specific to each farm studied, even on geographically close farms (from 1 to 73 km distance). Variations of MLVA profiles could be partially explained by sampling periods, since samples whom MLVA were interpretable were sampled between 1 day and 3 months apart depending of the farm. It has been reported a highest diversity of *Cryptosporidium* multi-loci genotypes circulating in cows than calves likely due to greater exposure from various origins for cows versus calves. Farms management practices and exposure to wildlife should also be considered [43].

Previously, increased oocyst shedding during parturition in cattle was demonstrated by microscopy. The authors suggested that this could play a significant role in contamination of newborn calves [44]. However, no genotyping data were available and calves were removed from their dams within 4 h and could also be contaminated by their environment [44]. Another study reported a majority of identical multilocus genotypes between calves and dams on beef farms, but a minority on dairy farms. The advanced explanation was that calves and dams were housed together on beef farms as opposed to dairy farms. They concluded that adult cattle (especially from dairy farms) are unlikely to play a significant role in the transmission of *Cryptosporidium* to calves [45], which is also suggested by our results.

*Cryptosporidium* is the most frequent digestive pathogen in newborn calves, although prevalence varies among the farms studied. Bovine rotavirus was the second most frequent. Systematic vaccination of dams could be effective in reducing symptomatology of calves. For the newly commercially available vaccine against cryptosporidiosis (Bovilis Cryptium®), further studies are needed to evaluate its efficacy under field conditions. For the investigation of cryptosporidiosis, PCR is more suitable for epidemiologic analysis, but antigenic testing offers a rapid response with acceptable performance in symptomatic calves. Among the *Cryptosporidium* species distribution, *C. parvum* dominates in both symptomatic and asymptomatic cattle, followed by *C. bovis*, *C. andersoni* and *C. ryanae*. *C. hominis* was detected in calves frequently exposed to humans, and *C. meleagridis* and *C. mortiferum* in symptomatic calves in rare cases. A large diversity of *C. parvum* subtypes (IIa and IId families) was observed between farms, with the highly transmissible IIaA15G2R1 subtype dominating. This IIaA15G2R1 subtype have widely been reported as the cause of outbreaks in both humans and animals from various origins (direct contact, water or food contamination). Results suggest that vertical transmission between calves and dams is unlikely to play a significant role in *Cryptosporidium* transmission route. MLVA profiles in calves appeared to be mainly herd-specific, even from geographically close farms.

## Supplementary Information

**Additional file 1. Detection of enteropathogens according to symptomatology of calves, sampling sites and vaccinal status.** Rotavirus / coronavirus / *E. coli* F5 / *E. coli* CS31A and “Ag *Cryptosporidium*” were detected by the Speed-V-Diar 5^©^ (Virbac Diagnostics). *Cryptosporidium* sp. were detected and identified by PCR.

**Additional file 2. Details of** ***Cryptosporidium*** **species and genotypes identification**. *Cryptosporidium* species and subtypes identification.

**Additional file 3. Details of** ***Cryptosporidium*** **species and genotypes identification according to calves and dams and sampling sites**. 0: Negative; NI: Not Identified (but positive for *Cryptosporidium* spp. DNA); NR: Not Realized.

## Data Availability

The datasets used and/or analysed during the current study are available from the corresponding author on reasonable request.
