# Supplementary material for: Prevalence of cryptosporidiosis and other enteric pathogens in calves in France: effects of rotavirus, coronavirus and E. coli vaccination and transmission routes
Source: Vet Res. 2026 Jun 9;57:103. doi: 10.1186/s13567-025-01609-6 (PMC13251175; doi:10.1186/s13567-025-01609-6)
Supplement: Supplementary file 1 — Additional file 1. Detection of enteropathogens according to symptomatology of calves, sampling sites and vaccinal status. Rotavirus / coronavirus / E. coli F5 / E. coli CS31A and “Ag Cryptosporidium” were detected by the Speed-V-Diar 5© (Virbac Diagnostics). Cryptosporidium sp. were detected and identified by PCR. [file 13567_2025_1609_MOESM1_ESM.doc]

**Additional file 1 Detection of enteropathogens according to symptomatology of calves, sampling sites and vaccinal status.** Rotavirus / coronavirus / *E. coli* F5 / *E. coli* CS31A and “Ag *Cryptosporidium*” were detected by the Speed-V-Diar 5© (Virbac Diagnostics). *Cryptosporidium* sp. were detected and identified by PCR.

Additional file 1 Distribution of enteropathogens in sampling sites. 0: Negative / 1: Positive / NR: Not Realized.

|  | **Enteropathogens detected from symptomatic calves in INRAE site** | | | | | | |
| --- | --- | --- | --- | --- | --- | --- | --- |
| **Calf number** | **Rotavirus** | **Coronavirus** | ***E. coli F5*** | ***E. Coli CS31A*** | **Ag *Cryptosporidium*** | ***Cryptosporidium sp.*** *(*gp60 *genotype when available)* | ***Dam vaccinated*** |
| FR6XXXXX9012 | 0 | 0 | 0 | 0 | 0 | *C. bovis* | *1* |
| FR6XXXXX9018 | 0 | 0 | 0 | 0 | 0 | *C. bovis* | *1* |
| FR6XXXXX9023 | 0 | 0 | 0 | 0 | 0 | 0 | *1* |
| FR6XXXXX9024 | 0 | 0 | 0 | 0 | 0 | 0 | *1* |
| FR6XXXXX9025 | 0 | 0 | 0 | 0 | 1 | IIaA15G2R1 | *1* |
| FR6XXXXX9026 | 0 | 0 | 0 | 0 | 0 | IIaA15G2R1 | *1* |
| FR6XXXXX9028 | 0 | 0 | 0 | 0 | 0 | 0 | *1* |
| FR6XXXXX9038 | 0 | 0 | 0 | 0 | 1 | IIaA15G2R1 | *1* |
| FR6XXXXX9050 | 0 | 0 | 0 | 0 | 1 | IIaA15G2R1 | *1* |
| FR6XXXXX9052 | 0 | 0 | 0 | 0 | 0 | IIaA15G2R1 | *1* |
| FR6XXXXX9070 | 0 | 0 | 0 | 0 | 0 | IIaA15G2R1 | *1* |
| FR6XXXXX9072 | 0 | 0 | 0 | 0 | 1 | IIaA15G2R1 | *1* |
| FR6XXXXX9074 | 0 | 0 | 0 | 0 | 0 | 0 | *1* |
| FR6XXXXX9093 | 0 | 0 | 0 | 0 | 1 | IIaA15G2R1 | *1* |
| FR6XXXXX9099 | 0 | 0 | 0 | 0 | 0 | IIaA15G2R1 | *1* |
| FR6XXXXX9100 | 0 | 0 | 0 | 0 | 1 | IIaA15G2R1 | *1* |
| FR6XXXXX9105 | 0 | 0 | 0 | 0 | 1 | IIaA15G2R1 | *1* |
| FR6XXXXX9106 | 0 | 0 | 0 | 0 | 1 | IIaA15G2R1 | *1* |
| FR6XXXXX9112 | 0 | 0 | 0 | 0 | 0 | IIaA15G2R1 | *1* |
| FR6XXXXX9114 | 0 | 0 | 0 | 0 | 1 | IIaA15G2R1 | *1* |
| FR6XXXXX9125 | 0 | 0 | 0 | 0 | 1 | IIaA15G2R1 | *1* |
| FR6XXXXX1023 | 0 | 0 | 0 | 0 | 0 | IIaA17G1R1 | *1* |
| FR6XXXXX1024 | 0 | 0 | 0 | 0 | 0 | 0 | *1* |
| FR6XXXXX2224 | 0 | 0 | 0 | 0 | 0 | 0 | *1* |
| FR6XXXXX2230 | 1 | 1 | 0 | 1 | 1 | NR | *1* |
| FR6XXXXX2234 | 1 | 1 | 1 | 1 | 1 | 0 | *1* |
| FR6XXXXX2235 | 1 | 1 | 1 | 1 | 1 | 0 | *1* |
| FR6XXXXX2250 | 0 | 0 | 0 | 0 | 0 | 0 | *1* |

|  | **Enteropathogens detected from asymptomatic calves in INRAE site** | | | | | | |
| --- | --- | --- | --- | --- | --- | --- | --- |
| **Calf number** | **Rotavirus** | **Coronavirus** | ***E. coli F5*** | ***E. Coli CS31A*** | **Ag *Cryptosporidium*** | ***Cryptosporidium sp.*** *(*gp60 *genotype when available)* | ***Dam vaccinated*** |
| FR6XXXXX1000 | 0 | 0 | 0 | 0 | 0 | IIdA18G1 | 1 |
| FR6XXXXX1001 | 0 | 0 | 0 | 0 | 0 | IIaA15G2R1 | 1 |
| FR6XXXXX1003 | 0 | 0 | 0 | 0 | 0 | IIdA25G3 | 1 |
| FR6XXXXX1004 | 0 | 0 | 0 | 0 | 0 | IIdA20G1 | 1 |
| FR6XXXXX1005 | 0 | 0 | 0 | 0 | 0 | 0 | 1 |
| FR6XXXXX1005 | 0 | 0 | 0 | 0 | 0 | IIaA17G2R1 | 1 |
| FR6XXXXX1006 | 0 | 0 | 0 | 0 | 0 | 0 | 1 |
| FR6XXXXX1007 | 0 | 0 | 0 | 0 | 0 | IIdA25G1 | 1 |
| FR6XXXXX1008 | 0 | 0 | 0 | 0 | 0 | 0 | 1 |
| FR6XXXXX1008 | 0 | 0 | 0 | 0 | 0 | 0 | 1 |
| FR6XXXXX1009 | 0 | 0 | 0 | 0 | 0 | 0 | 1 |
| FR6XXXXX1009 | 0 | 0 | 0 | 0 | 0 | 0 | 1 |
| FR6XXXXX1010 | 0 | 0 | 0 | 0 | 0 | IIaA15G2R1 | 1 |
| FR6XXXXX1011 | 0 | 0 | 0 | 0 | 0 | IIdA16G1 | 1 |
| FR6XXXXX1013 | 0 | 0 | 0 | 0 | 0 | IIaA17G1R1 | 1 |
| FR6XXXXX1014 | 0 | 0 | 0 | 0 | 0 | IIaA14G3R1 | 1 |
| FR6XXXXX1015 | 0 | 0 | 0 | 0 | 0 | 0 | 1 |
| FR6XXXXX1016 | 0 | 0 | 0 | 0 | 0 | IIaA18G5R1 /IIdA20 | 1 |
| FR6XXXXX1017 | 0 | 0 | 0 | 0 | 0 | IIaA15G2R1 | 1 |
| FR6XXXXX1018 | 0 | 0 | 0 | 0 | 0 | IIaA15G2R1 /IIdA21G2 /IIaA16G1R2 | 1 |
| FR6XXXXX1019 | 0 | 0 | 0 | 0 | 0 | IIaA15G2R1 / IIdA18G1 | 1 |
| FR6XXXXX1030 | 0 | 0 | 0 | 0 | 0 | 0 | 1 |
| FR6XXXXX1030 | 0 | 0 | 0 | 0 | 0 | IIdA18G1 | 1 |
| FR6XXXXX1031 | 0 | 0 | 0 | 0 | 0 | IIdA17G1 | 1 |
| FR6XXXXX1033 | 0 | 0 | 0 | 0 | 0 | IIdA17G1 | 1 |
| FR6XXXXX1035 | 0 | 0 | 0 | 0 | 0 | 0 | 1 |
| FR6XXXXX1037 | 0 | 0 | 0 | 0 | 0 | IIaA15G2R1 | 1 |
| FR6XXXXX1038 | 0 | 0 | 0 | 0 | 0 | IIdA21G1 | 1 |
| FR6XXXXX1039 | 0 | 0 | 0 | 0 | 0 | IIaA16G1R1 | 1 |
| FR6XXXXX1040 | 0 | 0 | 0 | 0 | 0 | IIaA17R1 | 1 |
| FR6XXXXX1041 | 0 | 0 | 0 | 0 | 0 | IIaA15G2R1 | 1 |
| FR6XXXXX1045 | 0 | 0 | 0 | 0 | 0 | 0 | 1 |
| FR6XXXXX1047 | 0 | 0 | 0 | 1 | 1 | IIaA18G1R1 | 1 |
| FR6XXXXX1056 | 0 | 0 | 0 | 0 | 0 | 0 | 1 |
| FR6XXXXX1057 | 0 | 0 | 0 | 0 | 0 | 0 | 1 |
| FR6XXXXX1060 | 0 | 0 | 0 | 0 | 0 | IIaA15G2R1 | 1 |
| FR6XXXXX1061 | 0 | 0 | 0 | 0 | 0 | 0 | 1 |
| FR6XXXXX1065 | 0 | 0 | 0 | 0 | 0 | IIaA15G2R1 | 1 |
| FR6XXXXX1066 | 0 | 0 | 0 | 0 | 0 | 0 | 1 |
| FR6XXXXX1067 | 0 | 0 | 0 | 0 | 0 | 0 | 1 |
| FR6XXXXX1068 | 0 | 0 | 0 | 0 | 0 | 0 | 1 |
| FR6XXXXX1074 | 0 | 0 | 0 | 0 | 0 | 0 | 1 |
| FR6XXXXX1076 | 0 | 0 | 0 | 0 | 0 | 0 | 1 |
| FR6XXXXX1077 | 0 | 0 | 0 | 0 | 0 | *C. bovis* | 1 |
| FR6XXXXX1080 | 0 | 0 | 0 | 0 | 0 | NR | 1 |
| FR6XXXXX1083 | 0 | 0 | 0 | 0 | 0 | NR | 1 |
| FR6XXXXX1084 | 0 | 0 | 0 | 0 | 0 | 0 | 1 |
| FR6XXXXX1087 | 0 | 0 | 0 | 0 | 0 | 0 | 1 |
| FR6XXXXX1088 | 0 | 0 | 0 | 0 | 0 | 0 | 1 |
| FR6XXXXX1090 | 0 | 0 | 0 | 0 | 0 | 0 | 1 |
| FR6XXXXX1095 | 1 | 0 | 0 | 0 | 0 | 0 | 1 |
| FR6XXXXX1098 | 0 | 0 | 0 | 0 | 0 | 0 | 1 |
| FR6XXXXX1100 | 1 | 0 | 0 | 0 | 0 | 0 | 1 |
| FR6XXXXX1100 | 0 | 0 | 0 | 0 | 0 | 0 | 1 |
| FR6XXXXX1101 | 0 | 0 | 0 | 1 | 0 | 0 | 1 |
| FR6XXXXX1104 | 0 | 0 | 0 | 0 | 0 | 0 | 1 |
| FR6XXXXX1109 | 0 | 0 | 0 | 0 | 0 | 0 | 1 |
| FR6XXXXX1110 | 0 | 0 | 0 | 0 | 0 | 0 | 1 |
| FR6XXXXX1110 | 0 | 0 | 0 | 0 | 0 | NR | 1 |
| FR6XXXXX1111 | 0 | 0 | 0 | 0 | 0 | 0 | 1 |
| FR6XXXXX1115 | 0 | 0 | 0 | 0 | 0 | NR | 1 |
| FR6XXXXX1117 | 0 | 0 | 0 | 0 | 0 | 0 | 1 |
| FR6XXXXX8350 | 0 | 0 | 0 | 0 | 0 | 0 | 1 |
| FR6XXXXX8355 | 0 | 0 | 0 | 0 | 0 | 0 | 1 |
| FR6XXXXX8357 | 0 | 0 | 0 | 0 | 0 | 0 | 1 |
| FR6XXXXX8358 | 0 | 0 | 0 | 0 | 0 | *C. ryanae* | 1 |
| FR6XXXXX8363 | 0 | 0 | 0 | 0 | 0 | 0 | 1 |
| FR6XXXXX8364 | 0 | 0 | 0 | 0 | 0 | 0 | 1 |
| FR6XXXXX8369 | 0 | 0 | 0 | 0 | 0 | 0 | 1 |
| FR6XXXXX8370 | 0 | 0 | 0 | 0 | 0 | *C. bovis* | 1 |
| FR6XXXXX8371 | 0 | 0 | 0 | 0 | 0 | *C. bovis* | 1 |
| FR6XXXXX8375 | 0 | 0 | 0 | 0 | 0 | 0 | 1 |
| FR6XXXXX8376 | 0 | 0 | 0 | 0 | 0 | 0 | 1 |
| FR6XXXXX8379 | 0 | 0 | 0 | 0 | 0 | 0 | 1 |
| FR6XXXXX8380 | 0 | 0 | 0 | 0 | 0 | 0 | 1 |
| FR6XXXXX8381 | 0 | 0 | 0 | 0 | 0 | 0 | 1 |
| FR6XXXXX8383 | 0 | 0 | 0 | 0 | 0 | 0 | 1 |
| FR6XXXXX8384 | 0 | 0 | 0 | 0 | 0 | 0 | 1 |
| FR6XXXXX8385 | 0 | 0 | 0 | 0 | 0 | 0 | 1 |
| FR6XXXXX8387 | 0 | 0 | 0 | 0 | 0 | 0 | 1 |
| FR6XXXXX8388 | 0 | 0 | 0 | 0 | 0 | 0 | 1 |
| FR6XXXXX9000 | 0 | 0 | 0 | 0 | 0 | 0 | 1 |
| FR6XXXXX9001 | 0 | 0 | 0 | 0 | 0 | 0 | 1 |
| FR6XXXXX9003 | 0 | 0 | 0 | 0 | 0 | 0 | 1 |
| FR6XXXXX9004 | 0 | 0 | 0 | 0 | 0 | 0 | 1 |
| FR6XXXXX9005 | 0 | 0 | 0 | 0 | 0 | 0 | 1 |
| FR6XXXXX9006 | 0 | 0 | 0 | 0 | 0 | 0 | 1 |
| FR6XXXXX9007 | 0 | 0 | 0 | 0 | 0 | IIaA20G1R1 | 1 |
| FR6XXXXX9008 | 0 | 0 | 0 | 0 | 0 | IIaA18G1R1 | 1 |
| FR6XXXXX9008 | 0 | 0 | 0 | 0 | 0 | 0 | 1 |
| FR6XXXXX9009 | 0 | 0 | 0 | 0 | 0 | 0 | 1 |
| FR6XXXXX9011 | 0 | 0 | 0 | 0 | 0 | *C. bovis* | 1 |
| FR6XXXXX9013 | 0 | 0 | 0 | 0 | 0 | 0 | 1 |
| FR6XXXXX9014 | 0 | 0 | 0 | 0 | 0 | 0 | 1 |
| FR6XXXXX9015 | 0 | 0 | 0 | 0 | 0 | *C. bovis* | 1 |
| FR6XXXXX9016 | 0 | 0 | 0 | 0 | 0 | IaA21R2 | 1 |
| FR6XXXXX9019 | 0 | 0 | 0 | 0 | 0 | IIaA15G2R1 | 1 |
| FR6XXXXX9030 | 0 | 0 | 0 | 0 | 1 | IIaA15G2R1 | 1 |
| FR6XXXXX9036 | 0 | 0 | 0 | 0 | 0 | IIaA15G2R1 | 1 |
| FR6XXXXX9040 | 0 | 0 | 0 | 0 | 0 | 0 | 1 |
| FR6XXXXX9041 | 0 | 0 | 0 | 0 | 1 | NR | 1 |
| FR6XXXXX9043 | 0 | 0 | 0 | 0 | 0 | IIaA15G2R1 | 1 |
| FR6XXXXX9044 | 0 | 0 | 0 | 0 | 0 | IIaA15G2R1 | 1 |
| FR6XXXXX9045 | 0 | 0 | 0 | 0 | 0 | IIaA15G2R1 | 1 |
| FR6XXXXX9046 | 0 | 0 | 0 | 0 | 0 | 0 | 1 |
| FR6XXXXX9048 | 0 | 0 | 0 | 0 | 0 | IIaA15G2R1 | 1 |
| FR6XXXXX9049 | 0 | 0 | 0 | 0 | 0 | IIaA15G2R1 | 1 |
| FR6XXXXX9053 | 0 | 0 | 0 | 0 | 0 | IIaA15G2R1 | 1 |
| FR6XXXXX9055 | 0 | 0 | 0 | 0 | 1 | IIaA15G2R1 | 1 |
| FR6XXXXX9060 | 0 | 0 | 0 | 0 | 0 | IIaA15G2R1 | 1 |
| FR6XXXXX9065 | 0 | 0 | 0 | 0 | 0 | IIaA15G2R1 | 1 |
| FR6XXXXX9069 | 0 | 0 | 0 | 0 | 0 | 0 | 1 |
| FR6XXXXX9070 | 0 | 0 | 0 | 0 | 0 | 0 | 1 |
| FR6XXXXX9074 | 0 | 0 | 0 | 0 | 1 | IIaA15G2R1 | 1 |
| FR6XXXXX9078 | 0 | 0 | 0 | 0 | 0 | IIaA15G2R1 | 1 |
| FR6XXXXX9079 | 0 | 0 | 0 | 0 | 0 | IIaA15G2R1 | 1 |
| FR6XXXXX9080 | 0 | 0 | 0 | 0 | 0 | 0 | 1 |
| FR6XXXXX9085 | 0 | 0 | 0 | 0 | 1 | IIaA15G2R1 | 1 |
| FR6XXXXX9087 | 0 | 0 | 1 | 0 | 1 | IIaA15G2R1 | 1 |
| FR6XXXXX9093 | 0 | 0 | 0 | 0 | 0 | 0 | 1 |
| FR6XXXXX9094 | 0 | 0 | 0 | 0 | 0 | IIaA15G2R1 | 1 |
| FR6XXXXX9096 | 0 | 0 | 0 | 0 | 0 | IIaA15G2R1 | 1 |
| FR6XXXXX9098 | 0 | 0 | 0 | 0 | 1 | IIaA15G2R1 | 1 |
| FR6XXXXX9103 | 0 | 0 | 1 | 0 | 0 | IIaA15G2R1 | 1 |
| FR6XXXXX9104 | 0 | 0 | 0 | 0 | 1 | IIaA15G2R1 | 1 |
| FR6XXXXX9109 | 0 | 0 | 0 | 0 | 0 | IIaA15G2R1 | 1 |
| FR6XXXXX9109 | 0 | 0 | 0 | 0 | 0 | 0 | 1 |
| FR6XXXXX9111 | 0 | 0 | 1 | 0 | 1 | IIaA15G2R1 | 1 |
| FR6XXXXX9118 | 0 | 1 | 0 | 0 | 1 | IIaA15G2R1 | 1 |
| FR6XXXXX9130 | 0 | 0 | 0 | 0 | 0 | 0 | 1 |
| FR6XXXXX9136 | 1 | 0 | 0 | 0 | 0 | IIaA15G2R1 | 1 |
| FR6XXXXX9138 | 1 | 0 | 0 | 0 | 1 | IIaA15G2R1 | 1 |
| FR6XXXXX9140 | 0 | 0 | 0 | 0 | 0 | IIaA15G2R1 | 1 |
| FR6XXXXX9146 | 1 | 0 | 0 | 0 | 0 | 0 | 1 |
| FR6XXXXX9147 | 0 | 0 | 0 | 0 | 0 | 0 | 1 |
| FR6XXXXX9149 | 0 | 0 | 0 | 0 | 0 | 0 | 1 |
| FR6XXXXX9151 | 0 | 0 | 0 | 0 | 0 | *C. parvum* | 1 |
| FR6XXXXX9154 | 0 | 0 | 0 | 0 | 0 | 0 | 1 |
| FR6XXXXX2218 | 0 | 0 | 0 | 0 | 0 | 0 | 1 |
| FR6XXXXX2219 | 0 | 0 | 0 | 0 | 0 | 0 | 1 |
| FR6XXXXX2220 | 0 | 0 | 0 | 0 | 0 | 0 | 1 |
| FR6XXXXX2221 | 0 | 0 | 0 | 0 | 0 | 0 | 1 |
| FR6XXXXX2222 | 0 | 0 | 0 | 0 | 0 | 0 | 1 |
| FR6XXXXX2223 | 0 | 0 | 0 | 0 | 0 | 0 | 1 |
| FR6XXXXX2225 | 0 | 0 | 0 | 0 | 0 | 0 | 1 |
| FR6XXXXX2226 | 0 | 0 | 0 | 0 | 0 | 0 | 1 |
| FR6XXXXX2227 | 0 | 0 | 0 | 0 | 0 | 0 | 1 |
| FR6XXXXX2228 | 0 | 1 | 1 | 1 | 0 | 0 | 1 |
| FR6XXXXX2229 | 0 | 0 | 0 | 0 | 0 | 0 | 1 |
| FR6XXXXX2231 | 0 | 1 | 0 | 0 | 0 | 0 | 1 |
| FR6XXXXX2232 | 0 | 0 | 0 | 0 | 0 | 0 | 1 |
| FR6XXXXX2233 | 0 | 0 | 0 | 0 | 0 | 0 | 1 |
| FR6XXXXX2236 | 1 | 1 | 1 | 1 | 1 | 0 | 1 |
| FR6XXXXX2237 | 1 | 1 | 1 | 1 | 1 | 0 | 1 |
| FR6XXXXX2238 | 0 | 0 | 0 | 0 | 0 | *C. bovis* | 1 |
| FR6XXXXX2239 | 0 | 0 | 0 | 0 | 0 | 0 | 1 |
| FR6XXXXX2240 | 0 | 0 | 0 | 0 | 0 | 0 | 1 |
| FR6XXXXX2241 | 0 | 0 | 0 | 0 | 0 | 0 | 1 |
| FR6XXXXX2242 | 0 | 0 | 0 | 0 | 0 | *C. bovis* | 1 |
| FR6XXXXX2243 | 1 | 1 | 1 | 0 | 1 | 0 | 1 |
| FR6XXXXX2244 | 0 | 0 | 0 | 0 | 0 | 0 | 1 |
| FR6XXXXX2245 | 0 | 0 | 0 | 0 | 0 | 0 | 1 |
| FR6XXXXX2246 | 0 | 0 | 0 | 0 | 0 | IbA10G2 / IIaA21G2R1 | 1 |
| FR6XXXXX2247 | 0 | 0 | 0 | 0 | 0 | 0 | 1 |
| FR6XXXXX2248 | 0 | 0 | 0 | 0 | 0 | *C. bovis* | 1 |
| FR6XXXXX2249 | 0 | 0 | 0 | 0 | 0 | IIaA14G2R1 | 1 |
| FR6XXXXX2251 | 0 | 0 | 0 | 0 | 0 | 0 | 1 |
| FR6XXXXX2252 | 1 | 1 | 1 | 1 | 1 | 0 | 1 |
| FR6XXXXX2253 | 0 | 1 | 0 | 0 | 0 | 0 | 1 |
| FR6XXXXX2254 | 1 | 1 | 1 | 1 | 1 | 0 | 1 |
| FR6XXXXX2256 | 1 | 1 | 1 | 0 | 1 | 0 | 1 |
| FR6XXXXX2258 | 0 | 0 | 0 | 0 | 0 | 0 | 1 |
| FR6XXXXX2259 | 0 | 0 | 0 | 0 | 0 | IIaA15G2R1 | 1 |
| FR6XXXXX2260 | 0 | 0 | 0 | 0 | 1 | 0 | 1 |
| FR6XXXXX2261 | 0 | 0 | 0 | 0 | 0 | 0 | 1 |
| FR6XXXXX2262 | 0 | 0 | 0 | 0 | 0 | 0 | 1 |
| FR6XXXXX2268 | 0 | 0 | 0 | 0 | 0 | 0 | 1 |
| FR6XXXXX2269 | 0 | 0 | 0 | 0 | 0 | IIaA14G3R1 | 1 |
| FR6XXXXX2272 | 0 | 0 | 0 | 0 | 0 | 0 | 1 |
| FR6XXXXX2274 | 0 | 0 | 0 | 0 | 0 | 0 | 1 |
| FR6XXXXX2276 | 0 | 0 | 0 | 0 | 0 | *C. bovis* | 1 |
| FR6XXXXX2277 | 0 | 0 | 0 | 0 | 0 | 0 | 1 |
| FR6XXXXX2282 | 0 | 0 | 0 | 0 | 0 | 0 | 1 |
| FR6XXXXX2290 | 1 | 1 | 1 | 0 | 1 | 0 | 1 |
| FR6XXXXX2296 | 0 | 0 | 0 | 0 | 0 | 0 | 1 |
| FR6XXXXX2306 | 0 | 0 | 0 | 0 | 0 | 0 | 1 |
| FR6XXXXX2308 | 0 | 0 | 0 | 0 | 0 | 0 | 1 |
| FR6XXXXX2312 | 0 | 0 | 0 | 0 | 0 | 0 | 1 |
| FR6XXXXX2316 | 0 | 0 | 0 | 0 | 0 | IIaA15G2R1 | 1 |
| FR6XXXXX2319 | 0 | 0 | 0 | 0 | 0 | IIaA15G2R1 | 1 |
| FR6XXXXX2323 | 0 | 0 | 0 | 0 | 0 | IIaA15G2R1 | 1 |
| FR6XXXXX2324 | 0 | 0 | 0 | 0 | 1 | IIaA15G2R1 | 1 |
| FR6XXXXX2326 | 1 | 0 | 1 | 0 | 1 | 0 | 1 |
| FR6XXXXX2327 | 0 | 0 | 0 | 0 | 0 | IIaA15G2R1 | 1 |
| FR6XXXXX2329 | 0 | 0 | 0 | 0 | 0 | IIaA15G2R1 | 1 |
| FR6XXXXX2330 | 0 | 0 | 0 | 1 | 1 | 0 | 1 |
| FR6XXXXX2331 | 0 | 0 | 0 | 0 | 0 | IIaA15G2R1 | 1 |
| FR6XXXXX2332 | 0 | 0 | 0 | 0 | 1 | IIaA15G2R1 | 1 |
| FR6XXXXX2335 | 0 | 0 | 0 | 0 | 0 | IIaA15G2R1 | 1 |
| FR6XXXXX2339 | 0 | 0 | 0 | 0 | 0 | 0 | 1 |
| FR6XXXXX2340 | 0 | 0 | 0 | 0 | 0 | 0 | 1 |
| FR6XXXXX2341 | 1 | 0 | 0 | 0 | 0 | 0 | 1 |
| FR6XXXXX2342 | 0 | 0 | 0 | 0 | 1 | 0 | 1 |
| FR6XXXXX2344 | 0 | 0 | 0 | 0 | 0 | 0 | 1 |
| FR6XXXXX2345 | 0 | 0 | 0 | 0 | 0 | 0 | 1 |
| FR6XXXXX2346 | 0 | 0 | 0 | 0 | 0 | 0 | 1 |
| FR6XXXXX2349 | 0 | 0 | 0 | 0 | 0 | 0 | 1 |
| FR6XXXXX2352 | 0 | 0 | 0 | 0 | 0 | 0 | 1 |
| FR6XXXXX2353 | 0 | 0 | 0 | 0 | 0 | *C. parvum* | 1 |
| FR6XXXXX2354 | 0 | 0 | 0 | 0 | 0 | *C. parvum* | 1 |
| FR6XXXXX2356 | 1 | 1 | 1 | 0 | 1 | 0 | 1 |
| FR6XXXXX2357 | 0 | 0 | 0 | 0 | 0 | 0 | 1 |
| FR6XXXXX2361 | 0 | 0 | 0 | 0 | 0 | 0 | 1 |

|  | **Enteropathogens detected from symptomatic calves in GDS sites** | | | | | |  |
| --- | --- | --- | --- | --- | --- | --- | --- |
| **Calf number** | **Rotavirus** | **Coronavirus** | ***E. coli F5*** | ***E. Coli CS31A*** | **Ag *Cryptosporidium*** | ***Cryptosporidium sp.*** *(*gp60 *genotype when available)* | ***Dam vaccinated*** |
| FR2XXXXX4682 | 1 | 0 | 0 | 1 | 0 | 0 | NR |
| FR2XXXXX4685 | 0 | 1 | 0 | 0 | 0 | 0 | NR |
| FR2XXXXX4686 | 1 | 0 | 0 | 0 | 0 | 0 | NR |
| FR2XXXXX1057 | 0 | 0 | 0 | 0 | 1 | IIaA15G2R1 | 0 |
| FR2XXXXX1058 | 0 | 0 | 0 | 0 | 1 | IIaA15G2R1 | 0 |
| FR2XXXXX4731 | 0 | 0 | 0 | 0 | 1 | IIaA15G2R1 | 0 |
| FR2XXXXX349 | 0 | 0 | 0 | 0 | 0 | 0 | 1 |
| FR22XXXXX074 | 0 | 1 | 0 | 0 | 1 | 0 | NR |
| FR2XXXXX23075 | 0 | 0 | 0 | 0 | 1 | IIaA15G2R1 | NR |
| FR2XXXXX3110 | 1 | 1 | 0 | 0 | 1 | IIaA15G2R1 | NR |
| FR2XXXXX3111 | 1 | 0 | 0 | 0 | 1 | IIaA15G2R1 | NR |
| FR2XXXXX8393 | 1 | 1 | 0 | 0 | 1 | IIaA15G2R1 | NR |
| FR2XXXXX5312 | 0 | 0 | 1 | 0 | 0 | 0 | NR |
| FR2XXXXX5318 | 0 | 0 | 1 | 0 | 0 | 0 | NR |
| FR2XXXXX5382 | 0 | 0 | 0 | 0 | 1 | IIaA15G2R1 | 0 |
| FR2XXXXX61925 | 0 | 0 | 0 | 0 | 0 | 0 | NR |
| FR2XXXXX61940 | 0 | 0 | 0 | 0 | 0 | IIaA20G1R1 | NR |
| FR2XXXXX61956 | 0 | 0 | 0 | 1 | 1 | IIaA15G2R1 | NR |
| FR2XXXXX61957 | 1 | 0 | 0 | 0 | 1 | IIaA15G2R1 | NR |
| FR2XXXXX53656 | 0 | 0 | 0 | 0 | 1 | C. parvum | 1 |
| FR2XXXXX53675 | 1 | 0 | 0 | 0 | 0 | IIaA15G2R1 | 1 |
| FR2XXXXX53684 | 0 | 1 | 0 | 0 | 1 | IIaA15G2R1 | 1 |
| FR2XXXXX96989 | 0 | 0 | 0 | 0 | 0 | IIaA15G2R1 | 1 |
| FR2XXXXX96995 | 1 | 0 | 0 | 0 | 1 | IIaA15G2R1 | 1 |
| FR2XXXXX96996 | 0 | 0 | 0 | 0 | 1 | IIaA15G2R1 | 1 |
| FR2XXXXX97005 | 0 | 0 | 0 | 0 | 1 | IIaA15G2R1 | 1 |
| FR2XXXXX97009 | 0 | 0 | 0 | 0 | 1 | IIaA15G2R1 | 1 |
| FR2XXXXX97012 | 0 | 0 | 0 | 0 | 1 | IIaA15G2R1 | 1 |
| FR2XXXXX97014 | 0 | 0 | 0 | 0 | 1 | *C. parvum* | *1* |
| FR2XXXXX7895 | 0 | 0 | 1 | 0 | 0 | IIaA15G2R1 | NR |
| FR29XXXXX259 | 0 | 0 | 0 | 1 | 0 | 0 | NR |
| FR2XXXXX261 | 0 | 0 | 1 | 1 | 0 | IIdA17G1 | NR |
| FR2XXXXX8173 | 1 | 0 | 0 | 0 | 1 | 0 | 0 |
| FR2XXXXX8175 | 0 | 0 | 0 | 0 | 0 | IIaA15G2R1 | 0 |
| FR29XXXXX176 | 1 | 0 | 0 | 0 | 1 | 0 | 0 |
| FR2XXXXX48177 | 0 | 0 | 0 | 0 | 1 | IIaA15G2R1 | 0 |
| FR2XXXXX48179 | 0 | 0 | 0 | 0 | 0 | IIaA15G2R1 | 0 |
| FR2XXXXX48189 | 0 | 0 | 0 | 0 | 1 | 0 | 0 |
| FR2XXXXX48191 | 0 | 0 | 0 | 0 | 0 | 0 | 0 |
| FR2XXXXX48195 | 0 | 0 | 0 | 0 | 1 | IIaA15G2R1 | 0 |
| FR2XXXXX48196 | 0 | 0 | 0 | 0 | 1 | IIaA15G2R1 | 0 |
| FR2XXXXX48197 | 1 | 0 | 0 | 0 | 1 | IIaA15G2R1 | 0 |
| FR2XXXXX48198 | 1 | 0 | 0 | 0 | 1 | IIaA15G2R1 | 0 |
| FR2XXXXX48200 | 0 | 0 | 0 | 0 | 1 | IIaA15G2R1 | 0 |
| FR2XXXXX48208 | 0 | 0 | 0 | 0 | 1 | IIaA15G2R1 | 0 |
| FR2XXXXX48213 | 0 | 0 | 0 | 1 | 0 | 0 | 0 |
| FR2XXXXX48223 | 0 | 0 | 0 | 0 | 1 | IIaA15G2R1 | 0 |
| FR2XXXXX48224 | 1 | 0 | 0 | 0 | 0 | 0 | 0 |
| FR2XXXXX48225 | 0 | 0 | 0 | 0 | 0 | 0 | 0 |
| FR2XXXXX48393 | 0 | 0 | 0 | 0 | 0 | IIaA15G2R1 | NR |
| FR2XXXXX48395 | 1 | 0 | 0 | 0 | 1 | IIaA15G2R1 | NR |
| FR2XXXXX48396 | 0 | 0 | 0 | 0 | 1 | IIaA15G2R1 | NR |
| FR2XXXXX48419 | 0 | 0 | 0 | 0 | 1 | IIaA15G2R1 | NR |
| FR2XXXXX48426 | 0 | 0 | 0 | 0 | 1 | IIaA15G2R1 | NR |
| FR2XXXXX48427 | 0 | 0 | 0 | 0 | 1 | IIaA15G2R1 | NR |
| FR2XXXXX48428 | 1 | 0 | 0 | 0 | 0 | 0 | NR |
| FR2XXXXX48431 | 1 | 0 | 0 | 0 | 1 | IIaA15G2R1 | NR |
| FR2XXXXX48432 | 1 | 0 | 0 | 0 | 1 | IIaA15G2R1 | NR |
| FR2XXXXX48446 | 1 | 0 | 0 | 0 | 0 | IIaA15G2R1 | NR |
| FR2XXXXX48447 | 1 | 0 | 0 | 0 | 0 | IIaA15G2R1 | NR |
| FR2XXXXX48449 | 0 | 0 | 0 | 0 | 1 | 0 | NR |
| FR2XXXXX48451 | 1 | 0 | 0 | 1 | 0 | IIaA15G2R1 | NR |
| FR2XXXXX48453 | 1 | 0 | 0 | 0 | 1 | IIaA15G2R1 | NR |
| FR2XXXXX48457 | 0 | 0 | 0 | 1 | 0 | IIaA15G2R1 | NR |
| FR2XXXXX48573 | 1 | 0 | 0 | 0 | 1 | IIaA15G2R1 | 0 |
| FR2XXXXX48575 | 1 | 1 | 0 | 0 | 1 | IIaA15G2R1 | 0 |
| FR2XXXXX48578 | 0 | 0 | 0 | 0 | 1 | 0 | 0 |
| FR2XXXXX48584 | 0 | 0 | 0 | 0 | 1 | *C. parvum* | *0* |
| FR2XXXXX48590 | 1 | 0 | 0 | 0 | 1 | 0 | 0 |
| FR2XXXXX48592 | 1 | 0 | 0 | 0 | 1 | *C. parvum* | *0* |
| FR2XXXXX48596 | 0 | 0 | 0 | 0 | 1 | IIaA15G2R1 | 0 |
| FR2XXXXX48597 | 1 | 0 | 0 | 0 | 1 | IIaA15G2R1 | 0 |
| FR2XXXXX48598 | 0 | 0 | 0 | 0 | 1 | IIaA15G2R1 | 0 |
| FR2XXXXX48600 | 1 | 0 | 0 | 0 | 0 | IIaA15G2R1 | 0 |
| FR2XXXXX48604 | 0 | 0 | 0 | 0 | 1 | 0 | 0 |
| FR2XXXXX48605 | 0 | 0 | 0 | 0 | 0 | *C. parvum* | *0* |
| FR2XXXXX48616 | 1 | 0 | 0 | 0 | 1 | IIaA15G2R1 | 0 |
| FR2XXXXX48617 | 0 | 0 | 0 | 0 | 1 | 0 | 0 |
| FR2XXXXX48620 | 0 | 0 | 0 | 0 | 1 | IIaA15G2R1 | 0 |
| FR2XXXXX48621 | 1 | 0 | 0 | 0 | 0 | IIaA15G2R1 | 0 |
| FR2XXXXX48624 | 1 | 0 | 0 | 0 | 1 | 0 | 0 |
| FR2XXXXX48625 | 1 | 0 | 0 | 0 | 1 | 0 | 0 |
| FR2XXXXX48627 | 1 | 0 | 0 | 0 | 1 | 0 | 0 |
| FR2XXXXX48629 | 0 | 0 | 0 | 0 | 0 | IIaA15G2R1 | 0 |
| FR2XXXXX48630 | 0 | 0 | 0 | 0 | 1 | IIaA15G2R1 | 0 |
| FR2XXXXX48631 | 1 | 0 | 0 | 0 | 1 | IIaA15G2R1 | 0 |
| FR2XXXXX48634 | 1 | 0 | 0 | 0 | 0 | IIaA15G2R1 | 0 |
| FR2XXXXX48635 | 1 | 0 | 0 | 0 | 0 | IIaA15G2R1 | 0 |
| FR2XXXXX48637 | 1 | 0 | 0 | 0 | 1 | 0 | 0 |
| FR2XXXXX35027 | 0 | 1 | 0 | 0 | 0 | IIaA15G2R1 | NR |
| FR2XXXXX35032 | 0 | 1 | 0 | 0 | 1 | IIaA15G2R1 | NR |
| FR2XXXXX35036 | 0 | 0 | 1 | 0 | 0 | 0 | NR |
| FR2XXXXX69649 | 1 | 0 | 0 | 0 | 1 | IIaA15G2R1 | NR |
| FR2XXXXX69656 | 0 | 1 | 0 | 0 | 1 | IIaA17G2R1 | NR |
| FR2XXXX68342 | 1 | 1 | 0 | 0 | 0 | 0 | 0 |
| FR2XXXXX6087 | 0 | 0 | 0 | 0 | 1 | IIaA15G2R1 | 0 |
| FR2XXXXX6091 | 1 | 0 | 0 | 0 | 0 | IIaA15G2R1 | 0 |
| FR2XXXXX69641 | 0 | 0 | 0 | 0 | 1 | IIaA15G2R1 | 0 |
| FR2XXXXX69646 | 0 | 0 | 0 | 0 | 1 | IIaA15G2R1 | 0 |
| FR2XXXXX69647 | 0 | 0 | 0 | 0 | 1 | IIaA15G2R1 | 0 |
| FR2XXXXX69648 | 0 | 0 | 0 | 0 | 1 | IIaA15G2R1 | 0 |
| FR2XXXXX69747 | 0 | 0 | 0 | 0 | 0 | IIaA15G2R1 | NR |
| FR2XXXXX69763 | 0 | 0 | 0 | 0 | 0 | 0 | NR |
| FR2XXXXX69767 | 0 | 1 | 0 | 0 | 0 | IIaA15G2R1 | NR |
| FR2XXXXX69771 | 1 | 0 | 0 | 0 | 0 | IIaA15G2R1 | NR |
| FR2XXXXX69773 | 1 | 1 | 0 | 0 | 1 | IIaA15G2R1 | NR |
| FR2XXXXX69774 | 1 | 0 | 0 | 0 | 0 | 0 | NR |
| FR2XXXXX69794 | 0 | 0 | 0 | 1 | 1 | IIaA15G2R1 | NR |
| FR2XXXX45719 | 1 | 0 | 0 | 0 | 0 | IIaA15G2R1 | NR |
| FR2XXXXX5743 | 0 | 0 | 1 | 0 | 1 | *C. bovis* | NR |
| FR2XXXX55052 | 0 | 0 | 0 | 0 | 0 | IIaA15G2R1 | 1 |
| FR2XXXX3662 | 0 | 0 | 0 | 0 | 0 | IIaA14G1R1 | NR |
| FR294XXXXXX4 | 0 | 0 | 0 | 1 | 1 | IIaA15G2R1 | NR |
| FR2XXXXX4901 | 0 | 0 | 0 | 0 | 0 | IIaA15G2R1 | NR |
| FR2XXXXX4903 | 0 | 0 | 0 | 0 | 0 | 0 | NR |
| FR2XXXXX4904 | 0 | 0 | 1 | 0 | 0 | 0 | NR |
| FR2XXXXX4955 | 0 | 0 | 0 | 0 | 0 | 0 | 0 |
| FR2XXXXX4958 | 0 | 0 | 0 | 0 | 0 | IIaA15G2R1 | 0 |
| FR2XXXXX5945 | 0 | 0 | 0 | 0 | 1 | 0 | 0 |
| FR2XXXXX5946 | 0 | 0 | 0 | 1 | 1 | IIaA15G2R1 | 0 |
| FR2XXXXX5954 | 0 | 0 | 0 | 0 | 1 | 0 | 0 |
| FR2XXXXX5994 | 0 | 1 | 0 | 1 | 1 | *C. parvum* | *0* |
| FR2XXXXX5998 | 0 | 1 | 0 | 0 | 1 | 0 | 0 |
| FR2XXXXX6000 | 1 | 0 | 0 | 0 | 1 | IIaA15G2R1 | 0 |
| FR2XXXXX2552 | 0 | 0 | 0 | 0 | 0 | IIaA15G2R1 | NR |
| FR2XXXXX10501 | 0 | 0 | 0 | 0 | 1 | IIaA15G2R1 | NR |
| FR2XXXXX10504 | 0 | 0 | 0 | 0 | 1 | IIaA15G2R1 | NR |
| FR2XXXXX10505 | 0 | 0 | 0 | 0 | 1 | IIaA15G2R1 | NR |
| FR2XXXXX10506 | 0 | 1 | 0 | 1 | 1 | IIaA15G2R1 | NR |
| FR2XXXXX10509 | 0 | 0 | 1 | 0 | 0 | 0 | NR |
| FR2XXXXX10511 | 0 | 0 | 1 | 0 | 0 | 0 | NR |
| FR2XXXXX10631 | 0 | 0 | 0 | 0 | 1 | *C. parvum* | *0* |
| FR2XXXXX10632 | 0 | 0 | 0 | 0 | 1 | *C. meleagridis* | *0* |
| FR2XXXXX10634 | 0 | 0 | 0 | 0 | 1 | IIaA15G2R1 | 0 |
| FR2XXXXX10636 | 0 | 0 | 0 | 0 | 1 | IIaA15G2R1 | 0 |
| FR2XXXXX10637 | 0 | 0 | 1 | 1 | 1 | IIaA15G2R1 | 0 |
| FR2XXXXX10640 | 1 | 0 | 0 | 0 | 1 | IIaA15G2R1 | 0 |
| FR2XXXXX10641 | 1 | 0 | 0 | 0 | 1 | IIaA15G2R1 | 0 |
| FR2XXXXX10643 | 1 | 0 | 0 | 0 | 1 | 0 | 0 |
| FR2XXXXX10651 | 0 | 0 | 0 | 0 | 1 | IIaA15G2R1 | 0 |
| FR2XXXXX10661 | 0 | 1 | 0 | 0 | 1 | IIaA15G2R1 | 0 |
| FR2XXXXX10662 | 1 | 0 | 0 | 1 | 0 | 0 | 0 |
| FR2XXXXX10663 | 1 | 1 | 0 | 0 | 1 | 0 | 0 |
| FR2XXXXX10664 | 1 | 0 | 0 | 0 | 0 | IIaA15G2R1 | 0 |
| FR2XXXXX10665 | 0 | 1 | 0 | 0 | 1 | *C. parvum* | *0* |
| FR2XXXXX84532 | 0 | 0 | 0 | 0 | 1 | IIaA15G2R1 | NR |
| FR2XXXXX84665 | 0 | 0 | 0 | 0 | 1 | IIaA15G2R1 | 0 |
| FR2XXXXX84666 | 0 | 0 | 0 | 0 | 1 | IIaA15G2R1 | 0 |
| FR2XXXXX84668 | 0 | 0 | 0 | 0 | 1 | IIaA15G2R1 | 0 |
| FR2XXXXX84673 | 0 | 0 | 0 | 1 | 1 | IIaA15G2R1 | 0 |
| FR2XXXXX84725 | 0 | 0 | 0 | 0 | 1 | IIaA15G2R1 | 0 |
| FR2XXXXX84730 | 0 | 0 | 0 | 0 | 1 | *C. parvum* | *0* |
| FR2XXXXX84734 | 0 | 0 | 0 | 0 | 0 | 0 | 0 |
| FR2XXXXX84735 | 0 | 0 | 0 | 0 | 0 | 0 | 0 |
| FR2XXXX2234 | 0 | 0 | 0 | 0 | 0 | 0 | NR |
| FR29XXXX926 | 0 | 0 | 0 | 0 | 1 | *C. bovis* | *1* |
| FR2XXXX3928 | 0 | 0 | 0 | 0 | 1 | *C. parvum* | *1* |
| FR2XXXXX13930 | 1 | 0 | 0 | 0 | 0 | 0 | 1 |
| FR2XXXXX13933 | 0 | 0 | 0 | 0 | 1 | IIaA15G2R1 | 1 |
| FR2XXXXX13934 | 0 | 1 | 0 | 0 | 1 | *C.parvum* | *1* |
| FR2XXXXX13942 | 0 | 0 | 0 | 0 | 0 | 0 | 1 |
| FR2XXXXX13945 | 0 | 0 | 0 | 0 | 1 | IIaA15G2R1 | 1 |
| FR2XXXXX13947 | 0 | 0 | 0 | 0 | 1 | *C.parvum* | *1* |
| FR2XXXXX13971 | 1 | 1 | 1 | 1 | 1 | 0 | 1 |
| FR2XXXXX13976 | 1 | 0 | 0 | 0 | 0 | 0 | 1 |
| FR2XXXXX13977 | 0 | 1 | 0 | 0 | 1 | 0 | 1 |
| FR2XXXXX13979 | 1 | 0 | 0 | 1 | 0 | IIaA15G2R1 | 1 |
| FR5XXXXX65828 | 0 | 0 | 0 | 0 | 0 | 0 | NR |
| FR5XXXXX65845 | 1 | 0 | 0 | 0 | 0 | IIaA15G2R1 | NR |
| FR5XXXXX65848 | 1 | 0 | 0 | 0 | 0 | IIaA15G2R1 | NR |
| FR5XXXXX2874 | 0 | 0 | 0 | 0 | 1 | IIaA15G2R1 | 1 |
| FR5XXXXX5935 | 0 | 0 | 0 | 0 | 1 | IIaA15G2R1 | NR |
| FR5XXXXX45832 | 0 | 0 | 0 | 0 | 0 | *C. mortiferum* | *0* |
| FR5XXXXX45833 | 1 | 0 | 0 | 0 | 1 | IIaA15G2R1 | 0 |
| FR5XXXXX45836 | 1 | 0 | 0 | 0 | 1 | IIaA15G2R1 | 0 |
| FR5XXXXX45837 | 0 | 0 | 0 | 0 | 0 | IIaA15G2R1 | 0 |
| FR5XXXXX23198 | 0 | 0 | 0 | 0 | 0 | IIaA15G2R1 | NR |
| FR5XXXXX23202 | 1 | 0 | 0 | 0 | 0 | IIaA15G2R1 | NR |
| FR5XXXXX23206 | 1 | 1 | 0 | 1 | 1 | IIaA15G2R1 | NR |
| FR5XXXXX23207 | 1 | 0 | 0 | 0 | 0 | IIaA15G2R1 | NR |
| FR5XXXXX23211 | 1 | 0 | 0 | 0 | 0 | IIaA15G2R1 | NR |
| FR5XXXXX23212 | 0 | 0 | 0 | 0 | 1 | IIaA15G2R1 | NR |
| FR5XXXXX23213 | 1 | 1 | 0 | 1 | 1 | IIaA15G2R1 | NR |
| FR5XXXXX23214 | 0 | 0 | 0 | 0 | 0 | IIaA15G2R1 | NR |

|  | **Enteropathogens detected from asymptomatic calves in GDS sites** | | | | | |  |
| --- | --- | --- | --- | --- | --- | --- | --- |
| **Calf number** | **Rotavirus** | **Coronavirus** | ***E. coli F5*** | ***E. Coli CS31A*** | **Ag *Cryptosporidium*** | ***Cryptosporidium sp.*** *(*gp60 *genotype when available)* | ***Dam vaccinated*** |
| FR2XXXXX4680 | 0 | 0 | 0 | 0 | 0 | IIaA19G1R1 | NR |
| FR2XXXXX4681 | 0 | 0 | 0 | 0 | 1 | IIaA19G1R1 | NR |
| FR2XXXXX1059 | 0 | 0 | 0 | 0 | 0 | IIaA15G2R1 | 0 |
| FR2XXXXX64732 | 1 | 0 | 1 | 0 | 0 | 0 | 0 |
| FR2XXXXX06350 | 0 | 0 | 0 | 0 | 0 | 0 | 1 |
| FR2XXXXX06358 | 0 | 0 | 0 | 0 | 0 | 0 | 1 |
| FR2XXXXX06359 | 0 | 0 | 0 | 0 | 0 | 0 | 1 |
| FR2XXXXX06360 | 0 | 0 | 0 | 0 | 0 | 0 | 1 |
| FR2XXXXX93723 | 0 | 0 | 0 | 0 | 0 | IIaA15G2R1 | 0 |
| FR2XXXXX93724 | 1 | 0 | 0 | 0 | 0 | 0 | 0 |
| FR2XXXXX93725 | 0 | 0 | 0 | 0 | 0 | 0 | 0 |
| FR2XXXXX93726 | 0 | 0 | 0 | 0 | 0 | IIaA15G2R1 | 0 |
| FR2XXXXX5308 | 0 | 0 | 0 | 0 | 0 | IIaA15G2R1 | NR |
| FR2XXXXX5383 | 0 | 1 | 0 | 1 | 1 | IIaA15G2R1 | 0 |
| FR2XXXX45384 | 0 | 0 | 0 | 1 | 0 | IIaA15G2R1 | 0 |
| FR2XXXXX5385 | 0 | 0 | 0 | 0 | 0 | IIaA15G2R1 | 0 |
| FR2XXXXX53660 | 0 | 0 | 0 | 1 | 1 | IIaA15G2R1 | 1 |
| FR2XXXXX53666 | 0 | 0 | 0 | 0 | 0 | 0 | 1 |
| FR2XXXXX53667 | 0 | 0 | 0 | 0 | 0 | 0 | 1 |
| FR2XXXXX53668 | 0 | 0 | 0 | 0 | 0 | 0 | 1 |
| FR2XXXXX53669 | 0 | 0 | 0 | 0 | 0 | IIaA15G2R1 | 1 |
| FR2XXXXX53670 | 0 | 0 | 0 | 0 | 0 | IIaA15G2R1 | 1 |
| FR2XXXXX53671 | 0 | 0 | 0 | 0 | 0 | 0 | 1 |
| FR2XXXXX53672 | 0 | 0 | 0 | 0 | 0 | 0 | 1 |
| FR2XXXXX53676 | 0 | 0 | 0 | 0 | 0 | 0 | 1 |
| FR2XXXXX53678 | 0 | 0 | 0 | 0 | 0 | 0 | 1 |
| FR2XXXXX53679 | 0 | 0 | 0 | 0 | 0 | 0 | 1 |
| FR2XXXXX53685 | 1 | 0 | 0 | 0 | 0 | IIaA15G2R1 | 1 |
| FR2XXXXX53686 | 0 | 0 | 0 | 0 | 1 | 0 | 1 |
| FR2XXXXX53687 | 0 | 0 | 0 | 0 | 0 | IIaA15G2R1 | 1 |
| FR2XXXXX53688 | 0 | 0 | 0 | 0 | 0 | 0 | 1 |
| FR2XXXXX96991 | 0 | 0 | 0 | 0 | 0 | IIaA15G2R1 | 1 |
| FR2XXXXX97008 | 0 | 0 | 0 | 0 | 1 | IIaA15G2R1 | 1 |
| FR2XXXXX97016 | 0 | 0 | 0 | 0 | 0 | 0 | 1 |
| FR2XXXXX257 | 0 | 0 | 0 | 0 | 0 | *C. bovis* | NR |
| FR2XXXXX2258 | 0 | 0 | 0 | 0 | 0 | *C. andersoni* | NR |
| FR29XXXXX260 | 0 | 0 | 0 | 0 | 0 | 0 | NR |
| FR2XXXXX48174 | 0 | 0 | 0 | 0 | 0 | IIaA15G2R1 | 0 |
| FR2XXXXX48181 | 0 | 0 | 0 | 0 | 0 | IIaA15G2R1 | 0 |
| FR2XXXXX48185 | 0 | 0 | 0 | 0 | 1 | 0 | 0 |
| FR2XXXXX48190 | 0 | 0 | 0 | 0 | 0 | 0 | 0 |
| FR2XXXXX48192 | 0 | 0 | 1 | 0 | 0 | 0 | 0 |
| FR2XXXXX48204 | 0 | 0 | 0 | 0 | 0 | IIaA15G2R1 | 0 |
| FR2XXXXX48205 | 0 | 0 | 0 | 0 | 0 | IIaA15G2R1 | 0 |
| FR2XXXXX48209 | 0 | 0 | 0 | 0 | 0 | IIaA15G2R1 | 0 |
| FR2XXXXX48210 | 0 | 0 | 0 | 0 | 0 | IIaA15G2R1 | 0 |
| FR2XXXXX48211 | 0 | 0 | 0 | 0 | 0 | 0 | 0 |
| FR2XXXXX48212 | 0 | 0 | 0 | 0 | 0 | 0 | 0 |
| FR2XXXXX48214 | 0 | 0 | 0 | 0 | 0 | IIaA15G2R1 | 0 |
| FR2XXXXX48215 | 0 | 0 | 0 | 0 | 0 | IIaA15G2R1 | 0 |
| FR2XXXXX48226 | 0 | 0 | 0 | 0 | 0 | 0 | 0 |
| FR2XXXXX48227 | 0 | 0 | 0 | 0 | 0 | 0 | 0 |
| FR2XXXXX48571 | 0 | 0 | 0 | 0 | 1 | 0 | 0 |
| FR2XXXXX48572 | 0 | 0 | 0 | 0 | 1 | IIaA15G2R1 | 0 |
| FR2XXXXX48574 | 1 | 0 | 0 | 0 | 1 | 0 | 0 |
| FR2XXXXX48576 | 1 | 0 | 0 | 0 | 0 | 0 | 0 |
| FR2XXXXX48577 | 1 | 0 | 0 | 0 | 0 | IIaA15G2R1 | 0 |
| FR2XXXXX48580 | 1 | 0 | 0 | 0 | 0 | *C. parvum* | *0* |
| FR2XXXXX48581 | 0 | 0 | 0 | 0 | 0 | IIaA15G2R1 | 0 |
| FR2XXXXX48582 | 0 | 0 | 0 | 0 | 0 | 0 | 0 |
| FR2XXXXX48583 | 0 | 0 | 0 | 0 | 0 | IIaA15G2R1 | 0 |
| FR2XXXXX48586 | 0 | 0 | 0 | 0 | 0 | IIaA15G2R1 | 0 |
| FR2XXXXX48587 | 0 | 0 | 0 | 0 | 0 | 0 | 0 |
| FR2XXXXX48591 | 1 | 0 | 0 | 0 | 0 | IIaA15G2R1 | 0 |
| FR2XXXXX48593 | 0 | 0 | 0 | 0 | 0 | 0 | 0 |
| FR2XXXXX48594 | 0 | 0 | 0 | 0 | 0 | 0 | 0 |
| FR2XXXXX48595 | 0 | 0 | 0 | 0 | 0 | 0 | 0 |
| FR2XXXXX48599 | 0 | 0 | 0 | 0 | 0 | IIaA15G2R1 | 0 |
| FR2XXXXX48601 | 0 | 0 | 0 | 0 | 0 | 0 | 0 |
| FR2XXXXX48602 | 0 | 0 | 0 | 0 | 1 | IIaA15G2R1 | 0 |
| FR2XXXXX48618 | 0 | 0 | 0 | 0 | 1 | IIaA15G2R1 | 0 |
| FR2XXXXX48626 | 1 | 0 | 0 | 0 | 0 | IIaA15G2R1 | 0 |
| FR2XXXXX48628 | 0 | 0 | 0 | 0 | 0 | IIaA15G2R1 | 0 |
| FR2XXXXX48633 | 1 | 0 | 0 | 0 | 1 | IIaA15G2R1 | 0 |
| FR2XXXXX48636 | 0 | 0 | 0 | 0 | 1 | *C. parvum* | *0* |
| FR2XXXXX48639 | 1 | 0 | 0 | 0 | 0 | 0 | 0 |
| FR2XXXXX68307 | 1 | 0 | 0 | 0 | 0 | *C. parvum* | *0* |
| FR2XXXXX68310 | 1 | 0 | 0 | 0 | 0 | 0 | 0 |
| FR2XXXXX68311 | 1 | 0 | 0 | 0 | 0 | 0 | 0 |
| FR2XXXXX68315 | 0 | 0 | 0 | 0 | 1 | IIaA15G2R1 | 0 |
| FR2XXXXX68317 | 0 | 0 | 0 | 0 | 0 | 0 | 0 |
| FR2XXXXX68318 | 1 | 0 | 0 | 0 | 0 | 0 | 0 |
| FR2XXXXX68319 | 0 | 0 | 0 | 0 | 1 | 0 | 0 |
| FR2XXXXX68325 | 1 | 0 | 0 | 0 | 0 | 0 | 0 |
| FR2XXXXX68326 | 0 | 0 | 0 | 0 | 0 | 0 | 0 |
| FR2XXXXX68329 | 1 | 0 | 0 | 0 | 0 | 0 | 0 |
| FR2XXXXX68330 | 0 | 0 | 0 | 0 | 0 | IIaA15G2R1 | 0 |
| FR2XXXXX68337 | 0 | 0 | 0 | 0 | 0 | 0 | 0 |
| FR2XXXXX68338 | 0 | 0 | 0 | 0 | 0 | IIaA15G2R1 | 0 |
| FR2XXXXX68341 | 0 | 0 | 0 | 0 | 1 | 0 | NR |
| FR2XXXXX68343 | 0 | 0 | 0 | 0 | 0 | IIaA15G2R1 | 0 |
| FR2XXXXX6086 | 0 | 0 | 0 | 0 | 1 | IIaA15G2R1 | 0 |
| FR2XXXXXX089 | 0 | 0 | 0 | 0 | 1 | IIaA15G2R1 | 0 |
| FR2XXXXX69644 | 0 | 0 | 0 | 0 | 1 | IIaA15G2R1 | 0 |
| FR2XXXXX69649 | 0 | 0 | 0 | 0 | 0 | 0 | 0 |
| FR2XXXXX69650 | 0 | 0 | 0 | 0 | 0 | IIaA15G2R1 | 0 |
| FR2XXXXX69748 | 0 | 0 | 0 | 0 | 0 | IIaA15G2R1 | NR |
| FR2XXXXX69754 | 0 | 0 | 0 | 0 | 0 | IIaA15G2R1 | NR |
| FR2XXXXX69756 | 0 | 0 | 0 | 0 | 0 | IIaA14G2R1 | NR |
| FR2XXXXX55048 | 0 | 0 | 0 | 0 | 0 | IIaA15G2R1 | 0 |
| FR2XXXXX55049 | 0 | 0 | 0 | 0 | 0 | 0 | 0 |
| FR2XXXXX55050 | 0 | 0 | 0 | 0 | 0 | 0 | 1 |
| FR2XXXXX55051 | 0 | 0 | 0 | 0 | 0 | IIaA15G2R1 | 1 |
| FR2XXXXX55053 | 0 | 0 | 0 | 0 | 0 | 0 | 1 |
| FR2XXXXX4894 | 0 | 0 | 0 | 0 | 0 | *C. bovis* | NR |
| FR2XXXXX4954 | 0 | 0 | 0 | 0 | 0 | IIaA15G2R1 | 0 |
| FR2XXXXX4957 | 0 | 0 | 0 | 0 | 0 | 0 | 0 |
| FR2XXXXX4959 | 0 | 0 | 0 | 0 | 0 | 0 | 0 |
| FR2XXXXX4960 | 0 | 0 | 0 | 0 | 0 | IIaA15G2R1 | 0 |
| FR2XXXXX5944 | 0 | 0 | 0 | 0 | 0 | 0 | 0 |
| FR2XXXXX5948 | 0 | 0 | 0 | 0 | 0 | 0 | 0 |
| FR2XXXXX5949 | 0 | 0 | 0 | 0 | 0 | IIaA15G2R1 | 0 |
| FR2XXXXX5951 | 0 | 0 | 0 | 0 | 1 | IIaA15G2R1 | 0 |
| FR2XXXXX5952 | 0 | 0 | 0 | 0 | 0 | IIaA15G2R1 | 0 |
| FR2XXXXX5953 | 0 | 0 | 0 | 0 | 0 | IIaA15G2R1 | 0 |
| FR2XXXXX5956 | 0 | 0 | 0 | 0 | 0 | 0 | 0 |
| FR2XXXXX5959 | 0 | 0 | 0 | 0 | 1 | IIaA15G2R1 | 0 |
| FR2XXXXX5960 | 0 | 0 | 0 | 0 | 0 | IIaA15G2R1 | 0 |
| FR2XXXXX5993 | 0 | 0 | 0 | 0 | 0 | IIaA15G2R1 | 0 |
| FR2XXXXX5995 | 0 | 0 | 0 | 0 | 0 | 0 | 0 |
| FR2XXXXX5996 | 0 | 0 | 0 | 0 | 0 | IIaA15G2R1 | 0 |
| FR2XXXXX5997 | 0 | 0 | 0 | 0 | 0 | IIaA15G2R1 | 0 |
| FR2XXXXX5999 | 0 | 0 | 0 | 0 | 0 | IIaA15G2R1 | 0 |
| FR2XXXXX10450 | 0 | 0 | 0 | 0 | 0 | IIaA15G2R1 | NR |
| FR2XXXXX10462 | 0 | 0 | 0 | 0 | 0 | 0 | NR |
| FR2XXXXX10624 | 0 | 0 | 0 | 0 | 0 | 0 | 0 |
| FR2XXXXX10626 | 0 | 0 | 0 | 0 | 1 | IIaA15G2R1 | 0 |
| FR2XXXXX10627 | 0 | 0 | 0 | 0 | 1 | IIaA15G2R1 | 0 |
| FR2XXXXX10628 | 0 | 0 | 0 | 0 | 0 | IIaA15G2R1 | 0 |
| FR2XXXXX10644 | 0 | 0 | 0 | 0 | 0 | IIaA15G2R1 | 0 |
| FR2XXXXX10650 | 0 | 0 | 0 | 0 | 0 | 0 | 0 |
| FR2XXXXX10652 | 0 | 0 | 0 | 0 | 1 | IIaA15G2R1 | 0 |
| FR2XXXXX10653 | 0 | 0 | 0 | 0 | 0 | 0 | 0 |
| FR2XXXXX10654 | 0 | 0 | 0 | 0 | 0 | *C. parvum* | *0* |
| FR2XXXXX10655 | 0 | 0 | 0 | 0 | 0 | IIaA15G2R1 | 0 |
| FR2XXXXX10657 | 0 | 0 | 0 | 0 | 0 | 0 | 0 |
| FR2XXXXX10658 | 0 | 0 | 0 | 0 | 0 | 0 | 0 |
| FR2XXXXX10659 | 0 | 0 | 0 | 0 | 0 | 0 | 0 |
| FR2XXXXX84623 | 0 | 0 | 0 | 0 | 0 | *C. andersoni* | *0* |
| FR2XXXXX84667 | 0 | 0 | 0 | 0 | 0 | IIaA15G2R1 | NR |
| FR2XXXXX84669 | 0 | 0 | 0 | 0 | 0 | IIaA15G2R1 / IIdA18G1 | 0 |
| FR2XXXXX84670 | 0 | 0 | 0 | 0 | 0 | 0 | 0 |
| FR2XXXXX84716 | 0 | 0 | 0 | 0 | 0 | IIaA15G2R1 | 0 |
| FR2XXXXX84723 | 0 | 0 | 0 | 0 | 0 | *C. parvum* | *0* |
| FR2XXXXX84726 | 0 | 0 | 0 | 0 | 0 | IIaA15G2R1 | 0 |
| FR2XXXXX84727 | 0 | 0 | 0 | 0 | 0 | IIaA15G2R1 | 0 |
| FR2XXXXX84736 | 1 | 0 | 0 | 0 | 0 | IIaA15G2R1 | 0 |
| FR2XXXXX84741 | 0 | 1 | 0 | 0 | 1 | 0 | 0 |
| FR2XXXXX84742 | 0 | 0 | 0 | 1 | 0 | IIaA15G2R1 | 0 |
| FR2XXXXX84744 | 0 | 0 | 0 | 0 | 0 | IIaA15G2R1 | 0 |
| FR2XXXXX84745 | 1 | 0 | 0 | 0 | 0 | IIaA15G2R1 | 0 |
| FR2XXXXXX231 | 0 | 0 | 0 | 0 | 0 | IIaA15G2R1 | NR |
| FR2XXXXX13929 | 0 | 0 | 0 | 0 | 0 | 0 | 1 |
| FR2XXXXX13931 | 1 | 0 | 0 | 1 | 0 | IIaA15G2R1 | 1 |
| FR2XXXXX13939 | 0 | 0 | 0 | 0 | 1 | 0 | 1 |
| FR2XXXXX13941 | 0 | 0 | 0 | 0 | 0 | IIaA15G2R1 | 1 |
| FR2XXXXX13969 | 0 | 1 | 0 | 0 | 1 | IIaA15G2R1 | 1 |
| FR2XXXXX13972 | 0 | 1 | 0 | 0 | 0 | 0 | 1 |
| FR2XXXXX13973 | 0 | 0 | 0 | 0 | 0 | IIaA15G2R1 | 1 |
| FR2XXXXX13980 | 0 | 0 | 0 | 0 | 0 | IIaA15G2R1 | 1 |
| FR5XXXXXX834 | 0 | 0 | 0 | 0 | 1 | IIaA15G2R1 | 0 |
| FR5XXXXX5835 | 0 | 0 | 0 | 0 | 0 | IIaA15G2R1 | 0 |
| FR5XXXXX65704 | 1 | 0 | 0 | 0 | 0 | IIaA15G2R1 | 0 |
| FR5XXXXX65705 | 0 | 0 | 0 | 0 | 0 | 0 | 0 |
| FR5XXXXX65743 | 0 | 0 | 0 | 0 | 0 | IIaA15G2R1 | 0 |
| FR5XXXXX32873 | 0 | 0 | 0 | 0 | 0 | IIaA15G2R1 | 1 |
| FR5XXXXX32875 | 0 | 0 | 0 | 0 | 0 | 0 | 1 |
| FR5XXXXX32877 | 0 | 0 | 0 | 0 | 0 | IIdA18G1 | 1 |
