# Supplementary material for: Prevalence of cryptosporidiosis and other enteric pathogens in calves in France: effects of rotavirus, coronavirus and E. coli vaccination and transmission routes
Source: Vet Res. 2026 Jun 9;57:103. doi: 10.1186/s13567-025-01609-6 (PMC13251175; doi:10.1186/s13567-025-01609-6)
Supplement: Supplementary file 2 — Additional file 2. Details of Cryptosporidium species and genotypes identification. Cryptosporidium species and subtypes identification. [file 13567_2025_1609_MOESM2_ESM.doc]

**Additional file 2 Details of *Cryptosporidium* species and genotypes identification**. *Cryptosporidium* species and subtypes identification.

|  | **Calves** | | **Cows** | **Calves** | | **Cows** |
| --- | --- | --- | --- | --- | --- | --- |
| **Cryptosporidium species** | **Symptomatic** | **Asymptomatic** | **Asymptomatic** | **Symptomatic** | **Asymptomatic** | **Asymptomatic** |
|  | **GDS** | | | **INRAE** | | |
| ***C. andersoni*** | / | 2 | 4 | / | / | 1 |
| ***C. bovis*** | 2 | 2 | 1 | 2 | 9 | 8 |
| ***C. ryanae*** | / | / | 2 | / | 1 | 1 |
| ***C. mortiferum*** | 1 | / | / | / | / | / |
| ***C. meleagridis*** | 1 | / | / | / | / | / |
| ***C. hominis and combination*** | | | | | | |
| ***C. hominis* IaA21R2** | / | / | / | / | 1 | / |
| ***C. hominis* IbA10G2 + *C. parvum* IIaA21G2R1** | / | / | / | / | 1 | / |
| ***C. hominis* IbA10G2 +C*.bovis*** | / | / | / | / | / | 1 |
| ***C. parvum and combination*** | | | | | | |
| ***C. parvum (without precise subtype)*** | 12 | 5 | 15 | / | 3 | 4 |
| **IIaA14G1R1** | 1 | / | / | / | / | / |
| **IIaA14G2R1** | / | 1 | 5 | / | 1 | / |
| **IIaA14G3R1** | / | / | / | / | 2 | / |
| **IIaA15G1R1** | / | / | 1 | / | / | 1 |
| **IIaA15G2R1** | 114 | 84 | 65 | 15 | 45 | 34 |
| **IIaA15G2R1+ IIdA18G1** | / | 1 | / | / | 1 | / |
| **IIaA15G2R1 + IIdA21G2 + IIaA16G1R2** | / | / | / | / | 1 | / |
| **IIaA16G1R1** | / | / | 1 | / | 1 | 2 |
| **IIaA16G3R1** | / | / | / | / | / | 1 |
| **IIaA17G1R1** | / | / | / | 1 | 1 | 1 |
| **IIaA17G2R1** | 1 | / | / | / | 1 | / |
| **IIaA17G3R1** | / | / | / | / | / | 1 |
| **IIaA17R1** | / | / | / | / | 1 | 1 |
| **IIaA18G1R1** | / | / | / | / | 2 | 1 |
| **IIaA18G5R1 + IIdA20** | / | / | / | / | 1 | / |
| **IIaA19G1R1** | / | 2 | / | / | / | 1 |
| **IIaA20G1R1** | 1 | / | / | / | 1 | 3 |
| **IIaA20R1** | / | / | / | / | / | 1 |
| **IIaA25G2R1** | / | / | 1 | / | / | / |
| **IIdA16G1** | / | / | / | / | 1 | / |
| **IIdA17G1** | 1 | / | / | / | 2 | / |
| **IIdA18G1** | / | 1 | 1 | / | 2 | 3 |
| **IIdA19** | / | / | / | / | / | 1 |
| **IIdA21G1** | / | / | / | / | 1 | / |
| **IIdA22G1** | / | / | / | / | 1 | / |
| **IIdA23G1** | / | / | / | / | / | / |
| **IIdA24G1** | / | / | / | / | / | 8 |
| **IIdA25G1** | / | / | / | / | 1 | / |
| **IIdA25G3** | / | / | / | / | 1 | / |
