# Supplementary material for: Prevalence of cryptosporidiosis and other enteric pathogens in calves in France: effects of rotavirus, coronavirus and E. coli vaccination and transmission routes
Source: Vet Res. 2026 Jun 9;57:103. doi: 10.1186/s13567-025-01609-6 (PMC13251175; doi:10.1186/s13567-025-01609-6)
Supplement: Supplementary file 3 — Additional file 3. Details of Cryptosporidium species and genotypes identification according to calves and dams and sampling sites. 0: Negative; NI: Not Identified (but positive for Cryptosporidium spp. DNA); NR: Not Realized. [file 13567_2025_1609_MOESM3_ESM.doc]

**Additional file 3 Details of *Cryptosporidium* species and genotypes identification according to calves and dams and sampling sites**. 0: Negative; NI: Not Identified (but positive for *Cryptosporidium* spp. DNA); NR: Not Realized.

.

| **Site** | **Dam genotype** | **Calf genotype** |
| --- | --- | --- |
| INRAE | 0 | 0 |
| INRAE | 0 | 0 |
| INRAE | 0 | IIaA15G2R1 |
| INRAE | 0 | IIaA15G2R1 |
| INRAE | 0 | IIaA15G2R1 |
| INRAE | 0 | 0 |
| INRAE | 0 | 0 |
| INRAE | 0 | IIaA15G2R1 |
| INRAE | 0 | IIaA15G2R1 |
| INRAE | 0 | 0 |
| INRAE | 0 | 0 |
| INRAE | 0 | NR |
| INRAE | 0 | IIaA15G2R1 |
| INRAE | 0 | IIaA15G2R1 |
| INRAE | 0 | IIaA15G2R1 |
| INRAE | 0 | IIaA15G2R1 |
| INRAE | 0 | 0 |
| INRAE | 0 | 0 |
| INRAE | 0 | 0 |
| INRAE | 0 | 0 |
| INRAE | 0 | 0 |
| INRAE | 0 | 0 |
| INRAE | 0 | 0 |
| INRAE | 0 | 0 |
| INRAE | 0 | 0 |
| INRAE | 0 | IIaA14G2R1 |
| INRAE | 0 | 0 |
| INRAE | 0 | IIaA15G2R1 |
| INRAE | 0 | IIaA15G2R1 |
| INRAE | 0 | 0 |
| INRAE | 0 | IbA10G2 / IIaA21G2R1 |
| INRAE | 0 | NR |
| INRAE | 0 | 0 |
| INRAE | 0 | IIaA15G2R1 |
| INRAE | 0 | IIaA17R1 |
| INRAE | 0 | 0 |
| INRAE | 0 | NA |
| INRAE | 0 | 0 |
| INRAE | 0 | NR |
| INRAE | 0 | NR |
| INRAE | 0 | NR |
| INRAE | 0 | IIaA15G2R1 |
| INRAE | 0 | IIaA15G2R1 |
| INRAE | 0 | 0 |
| INRAE | 0 | 0 |
| INRAE | 0 | 0 |
| INRAE | 0 | IIaA15G2R1 |
| INRAE | 0 | 0 |
| INRAE | 0 | NR |
| INRAE | 0 | NR |
| INRAE | 0 | IIaA17G1R1 |
| INRAE | 0 | 0 |
| INRAE | 0 | 0 |
| INRAE | 0 | 0 |
| INRAE | 0 | IaA21R2 |
| INRAE | 0 | 0 |
| INRAE | 0 | IIaA15G2R1 |
| INRAE | 0 | *C. bovis* |
| INRAE | 0 | 0 |
| INRAE | 0 | IIaA15G2R1 |
| INRAE | 0 | IIaA16G1R1 |
| INRAE | 0 | IIaA15G2R1 |
| INRAE | 0 | IIaA15G2R1 |
| INRAE | 0 | IIaA20G1R1 |
| INRAE | 0 | 0 |
| INRAE | 0 | 0 |
| INRAE | 0 | 0 |
| INRAE | 0 | 0 |
| INRAE | 0 | 0 |
| INRAE | 0 | 0 |
| INRAE | 0 | IIaA15G2R1 |
| INRAE | 0 | 0 |
| INRAE | 0 | IIaA15G2R1 |
| INRAE | 0 | 0 |
| INRAE | 0 | 0 |
| INRAE | 0 | 0 |
| INRAE | 0 | 0 |
| INRAE | 0 | IIdA25G1 |
| INRAE | 0 | IIdA17G1 |
| INRAE | 0 | 0 |
| INRAE | 0 | 0 |
| INRAE | 0 | 0 |
| INRAE | 0 | 0 |
| INRAE | 0 | 0 |
| INRAE | 0 | 0 |
| INRAE | 0 | IIdA16G1 |
| INRAE | 0 | IIdA21G1 |
| INRAE | 0 | 0 |
| INRAE | 0 | 0 |
| INRAE | 0 | 0 |
| INRAE | 0 | 0 |
| INRAE | 0 | 0 |
| INRAE | 0 | 0 |
| INRAE | 0 | 0 |
| INRAE | 0 | 0 |
| INRAE | 0 | 0 |
| INRAE | 0 | 0 |
| INRAE | 0 | 0 |
| INRAE | 0 | 0 |
| INRAE | 0 | 0 |
| INRAE | 0 | 0 |
| INRAE | 0 | 0 |
| INRAE | 0 | 0 |
| INRAE | 0 | 0 |
| INRAE | 0 | 0 |
| INRAE | 0 | *C. bovis* |
| INRAE | 0 | 0 |
| INRAE | 0 | *C. bovis* |
| INRAE | 0 | *C. bovis* |
| INRAE | 0 | 0 |
| INRAE | 0 | 0 |
| INRAE | 0 | 0 |
| INRAE | 0 | *C. parvum* |
| INRAE | 0 | 0 |
| INRAE | 0 | 0 |
| INRAE | 0 | 0 |
| INRAE | 0 | 0 |
| INRAE | 0 | 0 |
| INRAE | 0 | 0 |
| INRAE | 0 | 0 |
| INRAE | 0 | 0 |
| INRAE | 0 | *C. bovis* |
| INRAE | 0 | 0 |
| INRAE | 0 | IIaA14G3R1 |
| INRAE | 0 | IIaA15G2R1 |
| INRAE | 0 | 0 |
| INRAE | 0 | 0 |
| INRAE | 0 | 0 |
| INRAE | 0 | 0 |
| INRAE | 0 | 0 |
| INRAE | 0 | 0 |
| INRAE | 0 | *C. parvum* |
| INRAE | 0 | *C. bovis* |
| INRAE | 0 | 0 |
| INRAE | 0 | 0 |
| INRAE | 0 | *C. bovis* |
| INRAE | IIaA15G2R1 | IIaA18G1R1 |
| INRAE | *C. andersoni* | 0 |
| INRAE | *C. bovis* | IIaA15G2R1 |
| INRAE | *C. bovis* | IIaA15G2R1 |
| INRAE | *C. bovis* | 0 |
| INRAE | *C. bovis* | NR |
| INRAE | *C. bovis* | IIdA22G1 |
| INRAE | *C. bovis* | 0 |
| INRAE | *C. bovis* | 0 |
| INRAE | *C. bovis* / IbA10G2 | 0 |
| INRAE | *C. parvum* | IIaA15G2R1 |
| INRAE | *C. parvum* | IIaA15G2R1 |
| INRAE | *C. parvum* | IIaA15G2R1 |
| INRAE | *C. parvum* | IIaA15G2R1 |
| INRAE | *C. ryanae* | IIaA15G2R1 |
| INRAE | IIaA15G1R1 | 0 |
| INRAE | IIaA15G2R1 | IIaA15G2R1 |
| INRAE | IIaA15G2R1 | IIaA15G2R1 |
| INRAE | IIaA15G2R1 | IIaA17G1R1 |
| INRAE | IIaA15G2R1 | 0 |
| INRAE | IIaA15G2R1 | IIaA15G2R1 |
| INRAE | IIaA15G2R1 | IIaA15G2R1 |
| INRAE | IIaA15G2R1 | IIaA15G2R1 |
| INRAE | IIaA15G2R1 | IIaA15G2R1 |
| INRAE | IIaA15G2R1 | NR |
| INRAE | IIaA15G2R1 | 0 |
| INRAE | IIaA15G2R1 | 0 |
| INRAE | IIaA15G2R1 | IIaA15G2R1 |
| INRAE | IIaA15G2R1 | IIaA15G2R1 |
| INRAE | IIaA15G2R1 | IIaA15G2R1 |
| INRAE | IIaA15G2R1 | IIaA14G3R1 |
| INRAE | IIaA15G2R1 | 0 |
| INRAE | IIaA15G2R1 | IIaA15G2R1 / IIdA18G1 |
| INRAE | IIaA15G2R1 | IIaA15G2R1 |
| INRAE | IIaA15G2R1 | 0 |
| INRAE | IIaA15G2R1 | *C. bovis* |
| INRAE | IIaA15G2R1 | IIaA15G2R1 |
| INRAE | IIaA15G2R1 | 0 |
| INRAE | IIaA15G2R1 | 0 |
| INRAE | IIaA15G2R1 | IIdA25G3 |
| INRAE | IIaA15G2R1 | IIaA15G2R1 |
| INRAE | IIaA15G2R1 | 0 |
| INRAE | IIaA15G2R1 | 0 |
| INRAE | IIaA15G2R1 | IIaA15G2R1 |
| INRAE | IIaA15G2R1 | IIaA15G2R1 |
| INRAE | IIaA15G2R1 | IIaA15G2R1 |
| INRAE | IIaA15G2R1 | IIaA15G2R1 |
| INRAE | IIaA15G2R1 | IIaA15G2R1 |
| INRAE | IIaA15G2R1 | IIaA15G2R1 |
| INRAE | IIaA16G1R1 | IIaA15G2R1 |
| INRAE | IIaA16G1R1 | IIdA17G1 |
| INRAE | IIaA16G3R1 | *C. bovis* |
| INRAE | IIaA17G1R1 | IIaA15G2R1 |
| INRAE | IIaA17G3R1 | 0 |
| INRAE | IIaA17R1 | 0 |
| INRAE | IIaA18G1R1 | 0 |
| INRAE | IIaA19G1R1 | IIaA15G2R1 |
| INRAE | IIaA20G1R1 | *C. bovis* |
| INRAE | IIaA20G1R1 | 0 |
| INRAE | IIaA20G1R1 | 0 |
| INRAE | IIaA20R1 | IIaA15G2R1 |
| INRAE | IIdA18G1 | IIaA18G2R1 /IIdA20 |
| INRAE | IIdA18G1 | 0 |
| INRAE | IIdA18G1 | IIaA15G2R1 |
| INRAE | IIdA19 | IIaA15G2R1 /IIdA21G2 /IIaA16G1R2 |
| INRAE | NI | IIaA15G2R1 |
| INRAE | NI | IIaA15G2R1 |
| INRAE | NI | IIaA15G2R1 |
| INRAE | NI | IIaA15G2R1 |
| INRAE | NI | IIaA15G2R1 |
| INRAE | NI | *C. bovis* |
| INRAE | NI | IIaA15G2R1 |
| INRAE | NI | IIaA15G2R1 |
| INRAE | NI | 0 |
| INRAE | NI | IIaA15G2R1 |
| INRAE | NI | 0 |
| INRAE | NI | NR |
| INRAE | NI | C. bovis |
| INRAE | NI | 0 |
| INRAE | NI | IIaA15G2R1 |
| INRAE | NI | *C. bovis* |
| INRAE | NI | IIaA15G2R1 |
| INRAE | NI | IIaA15G2R1 |
| INRAE | NI | NR |
| INRAE | NI | *C. ryanae* |
| INRAE | NI | 0 |
| INRAE | NI | 0 |
| INRAE | NI | 0 |
| INRAE | NI | 0 |
| INRAE | NI | NR |
| INRAE | NI | NR |
| INRAE | NI | IIaA15G2R1 |
| INRAE | NI | IIaA15G2R1 |
| INRAE | NI | IIaA15G2R1 |
| INRAE | NI | IIaA15G2R1 |
| INRAE | NI | *C. parvum* |
| INRAE | NR | NR |
| INRAE | NR | NR |
| INRAE | NR | 0 |
| INRAE | NR | NR |
| INRAE | NR | 0 |
| INRAE | NR | NR |
|  |  |  |
| GDS | 0 | NR |
| GDS | 0 | NR |
| GDS | 0 | NR |
| GDS | 0 | IIaA15G2R1 |
| GDS | 0 | 0 |
| GDS | 0 | IIaA15G2R1 |
| GDS | 0 | 0 |
| GDS | 0 | 0 |
| GDS | 0 | NR |
| GDS | 0 | IIaA15G2R1 |
| GDS | 0 | IIaA15G2R1 |
| GDS | 0 | IIaA20G1R1 |
| GDS | 0 | IIaA17G2R1 |
| GDS | 0 | IIaA15G2R1 |
| GDS | 0 | IIdA17G1 |
| GDS | 0 | 0 |
| GDS | 0 | IIaA15G2R1 |
| GDS | 0 | NR |
| GDS | 0 | IIaA15G2R1 |
| GDS | 0 | IIaA15G2R1 |
| GDS | 0 | IIaA15G2R1 |
| GDS | 0 | IIaA15G2R1 |
| GDS | 0 | IIaA15G2R1 |
| GDS | 0 | NR |
| GDS | 0 | IIaA15G2R1 |
| GDS | 0 | IIaA15G2R1 |
| GDS | 0 | IIaA15G2R1 |
| GDS | 0 | IIaA15G2R1 |
| GDS | 0 | IIaA15G2R1 |
| GDS | 0 | IIaA15G2R1 |
| GDS | 0 | IIaA15G2R1 |
| GDS | 0 | IIaA15G2R1 |
| GDS | 0 | IIaA15G2R1 |
| GDS | 0 | NR |
| GDS | 0 | NR |
| GDS | 0 | IIaA15G2R1 |
| GDS | 0 | *C. bovis* |
| GDS | 0 | IIaA15G2R1 |
| GDS | 0 | NR |
| GDS | 0 | 0 |
| GDS | 0 | 0 |
| GDS | 0 | IIaA15G2R1 |
| GDS | 0 | IIaA15G2R1 |
| GDS | 0 | IIaA15G2R1 |
| GDS | 0 | 0 |
| GDS | 0 | IIaA15G2R1 |
| GDS | 0 | IIaA15G2R1 |
| GDS | 0 | IIaA15G2R1 |
| GDS | 0 | IIaA15G2R1 |
| GDS | 0 | IIaA15G2R1 |
| GDS | 0 | IIaA15G2R1 |
| GDS | 0 | IIaA15G2R1 |
| GDS | 0 | IIaA15G2R1 |
| GDS | 0 | NR |
| GDS | 0 | IIaA19G1R1 |
| GDS | 0 | IIaA15G2R1 |
| GDS | 0 | *C. bovis* |
| GDS | 0 | *C. andersoni* |
| GDS | 0 | 0 |
| GDS | 0 | NR |
| GDS | 0 | IIaA15G2R1 |
| GDS | 0 | IIaA14G2R1 |
| GDS | 0 | IIaA15G2R1 |
| GDS | 0 | NR |
| GDS | 0 | 0 |
| GDS | 0 | NR |
| GDS | 0 | NR |
| GDS | 0 | NR |
| GDS | 0 | *C. parvum* |
| GDS | 0 | IIaA15G2R1 |
| GDS | 0 | IIaA15G2R1 |
| GDS | 0 | IIaA15G2R1 |
| GDS | 0 | IIaA15G2R1 |
| GDS | 0 | *C. parvum* |
| GDS | 0 | IIaA15G2R1 |
| GDS | 0 | IIaA15G2R1 |
| GDS | 0 | IIaA15G2R1 |
| GDS | 0 | *C.parvum* |
| GDS | 0 | *C. parvum* |
| GDS | 0 | *C. bovis* |
| GDS | 0 | IIaA15G2R1 |
| GDS | 0 | 0 |
| GDS | 0 | 0 |
| GDS | 0 | 0 |
| GDS | 0 | IIaA15G2R1 |
| GDS | 0 | 0 |
| GDS | 0 | 0 |
| GDS | 0 | NA |
| GDS | 0 | IIaA15G2R1 |
| GDS | 0 | IIaA15G2R1 |
| GDS | 0 | IIaA15G2R1 |
| GDS | 0 | 0 |
| GDS | 0 | IIaA15G2R1 |
| GDS | 0 | 0 |
| GDS | 0 | 0 |
| GDS | 0 | 0 |
| GDS | 0 | 0 |
| GDS | 0 | NR |
| GDS | 0 | NR |
| GDS | 0 | IIaA15G2R1 |
| GDS | 0 | NR |
| GDS | 0 | 0 |
| GDS | 0 | 0 |
| GDS | 0 | NR |
| GDS | 0 | NR |
| GDS | 0 | NR |
| GDS | 0 | NR |
| GDS | 0 | 0 |
| GDS | 0 | 0 |
| GDS | 0 | 0 |
| GDS | 0 | IIaA15G2R1 |
| GDS | 0 | NR |
| GDS | 0 | IIaA15G2R1 |
| GDS | 0 | NR |
| GDS | 0 | NR |
| GDS | 0 | IIaA15G2R1 |
| GDS | 0 | 0 |
| GDS | 0 | 0 |
| GDS | 0 | IIaA15G2R1 |
| GDS | 0 | IIaA15G2R1 |
| GDS | 0 | IIaA15G2R1 |
| GDS | 0 | IIaA15G2R1 |
| GDS | 0 | IIdA18G1 |
| GDS | 0 | NA |
| GDS | 0 | IIaA15G2R1 |
| GDS | 0 | IIaA15G2R1 |
| GDS | 0 | IIaA15G2R1 |
| GDS | 0 | IIaA15G2R1 |
| GDS | 0 | IIaA15G2R1 |
| GDS | 0 | 0 |
| GDS | 0 | 0 |
| GDS | 0 | IIaA15G2R1 |
| GDS | 0 | 0 |
| GDS | 0 | *C. parvum* |
| GDS | 0 | IIaA15G2R1 |
| GDS | 0 | 0 |
| GDS | 0 | *C. parvum* |
| GDS | 0 | IIaA15G2R1 |
| GDS | 0 | *C. parvum* |
| GDS | 0 | 0 |
| GDS | 0 | IIaA15G2R1 |
| GDS | 0 | IIaA15G2R1 |
| GDS | 0 | IIaA15G2R1 |
| GDS | 0 | 0 |
| GDS | 0 | IIaA15G2R1 |
| GDS | 0 | IIaA15G2R1 |
| GDS | 0 | 0 |
| GDS | 0 | 0 |
| GDS | 0 | *C. parvum* |
| GDS | 0 | IIaA15G2R1 |
| GDS | 0 | IIaA15G2R1 |
| GDS | 0 | 0 |
| GDS | 0 | IIaA15G2R1 |
| GDS | 0 | 0 |
| GDS | 0 | IIaA15G2R1 |
| GDS | 0 | IIaA15G2R1 |
| GDS | 0 | 0 |
| GDS | 0 | *C. meleagridis* |
| GDS | 0 | IIaA15G2R1 |
| GDS | 0 | IIaA15G2R1 |
| GDS | 0 | IIaA15G2R1 |
| GDS | 0 | IIaA15G2R1 |
| GDS | 0 | *C. parvum* |
| GDS | 0 | IIaA15G2R1 |
| GDS | 0 | IIaA15G2R1 |
| GDS | 0 | IIaA15G2R1 |
| GDS | 0 | 0 |
| GDS | 0 | 0 |
| GDS | 0 | NR |
| GDS | 0 | IIaA15G2R1 |
| GDS | 0 | NR |
| GDS | 0 | 0 |
| GDS | 0 | IIaA15G2R1 |
| GDS | 0 | IIaA15G2R1 |
| GDS | 0 | IIaA15G2R1 |
| GDS | 0 | 0 |
| GDS | 0 | 0 |
| GDS | 0 | IIaA15G2R1 |
| GDS | 0 | IIaA15G2R1 |
| GDS | 0 | IIaA15G2R1 |
| GDS | 0 | 0 |
| GDS | 0 | IIaA15G2R1 |
| GDS | 0 | *C. parvum* |
| GDS | 0 | IIaA15G2R1 |
| GDS | 0 | IIaA15G2R1 |
| GDS | 0 | IIaA15G2R1 |
| GDS | 0 | 0 |
| GDS | 0 | IIaA15G2R1 |
| GDS | 0 | 0 |
| GDS | 0 | 0 |
| GDS | 0 | NR |
| GDS | 0 | NR |
| GDS | 0 | 0 |
| GDS | 0 | 0 |
| GDS | 0 | C. parvum |
| GDS | 0 | 0 |
| GDS | 0 | IIaA15G2R1 |
| GDS | 0 | 0 |
| GDS | 0 | 0 |
| GDS | 0 | IIaA15G2R1 |
| GDS | 0 | 0 |
| GDS | 0 | IIaA15G2R1 |
| GDS | 0 | IIaA15G2R1 |
| GDS | 0 | IIaA15G2R1 |
| GDS | 0 | NR |
| GDS | 0 | IIaA15G2R1 |
| GDS | 0 | 0 |
| GDS | 0 | IIaA15G2R1 |
| GDS | 0 | IIaA15G2R1 |
| GDS | 0 | 0 |
| GDS | 0 | 0 |
| GDS | 0 | NR |
| GDS | 0 | NR |
| GDS | 0 | IIaA15G2R1 |
| GDS | 0 | IIaA15G2R1 |
| GDS | 0 | 0 |
| GDS | 0 | NR |
| GDS | 0 | IIaA15G2R1 |
| GDS | 0 | IIaA15G2R1 |
| GDS | 0 | IIaA15G2R1 |
| GDS | 0 | 0 |
| GDS | 0 | 0 |
| GDS | 0 | NR |
| GDS | 0 | NR |
| GDS | 0 | 0 |
| GDS | 0 | 0 |
| GDS | 0 | NR |
| GDS | 0 | IIaA15G2R1 |
| GDS | 0 | *C. parvum* |
| GDS | 0 | IIaA15G2R1 |
| GDS | 0 | IIaA15G2R1 |
| GDS | 0 | IIaA15G2R1 |
| GDS | 0 | IIaA15G2R1 |
| GDS | 0 | NR |
| GDS | 0 | IIaA15G2R1 |
| GDS | 0 | *C. parvum* |
| GDS | 0 | IIaA15G2R1 |
| GDS | 0 | 0 |
| GDS | 0 | IIaA15G2R1 |
| GDS | 0 | IIaA15G2R1 |
| GDS | 0 | IIaA15G2R1 |
| GDS | 0 | NR |
| GDS | 0 | IIaA15G2R1 |
| GDS | 0 | IIaA15G2R1 / IIdA18G1 |
| GDS | 0 | IIaA15G2R1 |
| GDS | 0 | IIaA15G2R1 |
| GDS | 0 | 0 |
| GDS | 0 | IIaA15G2R1 |
| GDS | 0 | 0 |
| GDS | 0 | IIaA15G2R1 |
| GDS | 0 | IIaA15G2R1 |
| GDS | *C. andersoni* | IIaA15G2R1 |
| GDS | *C. andersoni* | NR |
| GDS | *C. andersoni* | 0 |
| GDS | *C. andersoni* | IIaA15G2R1 |
| GDS | *C. bovis* | IIaA15G2R1 |
| GDS | *C. parvum* | IIaA15G2R1 |
| GDS | *C. parvum* | 0 |
| GDS | *C. parvum* | 0 |
| GDS | *C. parvum* | IIaA15G2R1 |
| GDS | *C. parvum* | 0 |
| GDS | *C. parvum* | 0 |
| GDS | *C. parvum* | IIaA15G2R1 |
| GDS | *C. parvum* | IIaA15G2R1 |
| GDS | *C. parvum* | IIaA15G2R1 |
| GDS | *C. parvum* | IIaA15G2R1 |
| GDS | *C. parvum* | IIaA15G2R1 |
| GDS | *C. parvum* | IIaA15G2R1 |
| GDS | *C. parvum* | 0 |
| GDS | *C. parvum* | IIaA15G2R1 |
| GDS | *C. ryanae* | 0 |
| GDS | *C. ryanae* | IIaA15G2R1 |
| GDS | *C.parvum* | 0 |
| GDS | IIaA14G2R1 | IIaA15G2R1 |
| GDS | IIaA14G2R1 | 0 |
| GDS | IIaA14G2R1 | IIaA15G2R1 |
| GDS | IIaA14G2R1 | NR |
| GDS | IIaA14G2R1 | IIaA15G2R1 |
| GDS | IIaA15G1R1 | IIaA15G2R1 |
| GDS | IIaA15G2R1 | IIaA15G2R1 |
| GDS | IIaA15G2R1 | IIaA14G1R1 |
| GDS | IIaA15G2R1 | IIaA15G2R1 |
| GDS | IIaA15G2R1 | IIaA15G2R1 |
| GDS | IIaA15G2R1 | IIaA19G1R1 |
| GDS | IIaA15G2R1 | NR |
| GDS | IIaA15G2R1 | IIaA15G2R1 |
| GDS | IIaA15G2R1 | IIaA15G2R1 |
| GDS | IIaA15G2R1 | 0 |
| GDS | IIaA15G2R1 | *C.parvum* |
| GDS | IIaA15G2R1 | 0 |
| GDS | IIaA15G2R1 | NR |
| GDS | IIaA15G2R1 | IIaA15G2R1 |
| GDS | IIaA15G2R1 | IIaA15G2R1 |
| GDS | IIaA15G2R1 | 0 |
| GDS | IIaA15G2R1 | 0 |
| GDS | IIaA15G2R1 | 0 |
| GDS | IIaA15G2R1 | 0 |
| GDS | IIaA15G2R1 | IIaA15G2R1 |
| GDS | IIaA15G2R1 | IIaA15G2R1 |
| GDS | IIaA15G2R1 | 0 |
| GDS | IIaA15G2R1 | IIaA15G2R1 |
| GDS | IIaA15G2R1 | IIaA15G2R1 |
| GDS | IIaA15G2R1 | 0 |
| GDS | IIaA15G2R1 | 0 |
| GDS | IIaA15G2R1 | IIaA15G2R1 |
| GDS | IIaA15G2R1 | IIaA15G2R1 |
| GDS | IIaA15G2R1 | *C. parvum* |
| GDS | IIaA15G2R1 | IIaA15G2R1 |
| GDS | IIaA15G2R1 | IIaA15G2R1 |
| GDS | IIaA15G2R1 | *C. parvum* |
| GDS | IIaA15G2R1 | IIaA15G2R1 |
| GDS | IIaA15G2R1 | IIaA15G2R1 |
| GDS | IIaA15G2R1 | 0 |
| GDS | IIaA15G2R1 | IIaA15G2R1 |
| GDS | IIaA15G2R1 | IIaA15G2R1 |
| GDS | IIaA15G2R1 | IIaA15G2R1 |
| GDS | IIaA15G2R1 | IIaA15G2R1 |
| GDS | IIaA15G2R1 | 0 |
| GDS | IIaA15G2R1 | IIaA15G2R1 |
| GDS | IIaA15G2R1 | IIaA15G2R1 |
| GDS | IIaA15G2R1 | IIaA15G2R1 |
| GDS | IIaA15G2R1 | 0 |
| GDS | IIaA15G2R1 | IIaA15G2R1 |
| GDS | IIaA15G2R1 | IIaA15G2R1 |
| GDS | IIaA15G2R1 | 0 |
| GDS | IIaA15G2R1 | 0 |
| GDS | IIaA15G2R1 | IIaA15G2R1 |
| GDS | IIaA15G2R1 | 0 |
| GDS | IIaA15G2R1 | *C.parvum* |
| GDS | IIaA15G2R1 | 0 |
| GDS | IIaA15G2R1 | 0 |
| GDS | IIaA15G2R1 | 0 |
| GDS | IIaA15G2R1 | IIaA15G2R1 |
| GDS | IIaA15G2R1 | NR |
| GDS | IIaA15G2R1 | NR |
| GDS | IIaA15G2R1 | IIaA15G2R1 |
| GDS | IIaA15G2R1 | IIaA15G2R1 |
| GDS | IIaA15G2R1 | IIaA15G2R1 |
| GDS | IIaA15G2R1 | 0 |
| GDS | IIaA15G2R1 | 0 |
| GDS | IIaA15G2R1 | IIaA15G2R1 |
| GDS | IIaA15G2R1 | IIaA15G2R1 |
| GDS | IIaA15G2R1 | 0 |
| GDS | IIaA15G2R1 | IIaA15G2R1 |
| GDS | IIaA16G1R1 | IIaA15G2R1 |
| GDS | IIaA25G2R1 | NA |
| GDS | IIdA18G1 | IIaA15G2R1 |
| GDS | NI | 0 |
| GDS | NI | NA |
| GDS | NI | 0 |
| GDS | NI | NA |
| GDS | NI | IIaA15G2R1 |
| GDS | NI | IIaA15G2R1 |
| GDS | NI | IIaA15G2R1 |
| GDS | NI | NA |
| GDS | NI | 0 |
| GDS | NI | NA |
| GDS | NI | IIaA15G2R1 |
| GDS | NI | 0 |
| GDS | NI | IIaA15G2R1 |
| GDS | NI | IIaA15G2R1 |
| GDS | NI | *C. erinacei* |
| GDS | NI | IIaA15G2R1 |
| GDS | NI | 0 |
| GDS | NI | 0 |
| GDS | NI | IIaA15G2R1 |
| GDS | NI | IIaA15G2R1 |
| GDS | NI | IIaA15G2R1 |
| GDS | NI | NA |
| GDS | NI | IIaA15G2R1 |
| GDS | NI | IIaA15G2R1 |
| GDS | NI | IIaA15G2R1 |
| GDS | NI | NA |
| GDS | NI | NA |
| GDS | NI | IIaA15G2R1 |
| GDS | NI | 0 |
| GDS | NI | NA |
| GDS | NI | IIaA15G2R1 |
| GDS | NI | IIaA15G2R1 |
| GDS | NR | IIaA15G2R1 |
| GDS | NR | IIaA15G2R1 |
| GDS | NR | IIaA15G2R1 |
| GDS | NR | IIaA15G2R1 |
| GDS | NR | 0 |
| GDS | NR | *C. bovis* |
| GDS | NR | *C. andersoni* |
| GDS | NR | IIaA15G2R1 |
| GDS | NR | IIaA15G2R1 |
| GDS | NR | IIaA15G2R1 |
| GDS | NR | IIaA15G2R1 |
| GDS | NR | 0 |
| GDS | NR | 0 |
| GDS | NR | IIaA15G2R1 |
| GDS | NR | 0 |
| GDS | NR | NA |
